# Nectar biosynthesis is conserved among floral and extrafloral nectaries

**DOI:** 10.1093/plphys/kiab018

**Published:** 2021-01-28

**Authors:** Elizabeth C Chatt, Siti-Nabilla Mahalim, Nur-Aziatull Mohd-Fadzil, Rahul Roy, Peter M Klinkenberg, Harry T Horner, Marshall Hampton, Clay J Carter, Basil J Nikolau

**Affiliations:** 1 Roy J. Carver Department of Biochemistry, Biophysics and Molecular Biology, Iowa State University, Ames, 50010, Iowa; 2 Department of Plant and Microbial Biology, University of Minnesota Twin Cities, St. Paul, 55108, Minnesota; 3 Department of Biology, St. Catherine University, St. Paul, 55105, Minnesota; 4 Department of Genetics, Development and Cell Biology, Iowa State University, Ames, 50010, Iowa; 5 Roy J. Carver High Resolution Microscopy Facility, Iowa State University, Ames, 50010, Iowa; 6 Department of Mathematics and Statistics, University of Minnesota Duluth, Duluth, 55812, Minnesota

## Abstract

Nectar is a primary reward mediating plant–animal mutualisms to improve plant fitness and reproductive success. Four distinct trichomatic nectaries develop in cotton (*Gossypium hirsutum*), one floral and three extrafloral, and the nectars they secrete serve different purposes. Floral nectar attracts bees for promoting pollination, while extrafloral nectar attracts predatory insects as a means of indirect protection from herbivores. Cotton therefore provides an ideal system for contrasting mechanisms of nectar production and nectar composition between different nectary types. Here, we report the transcriptome and ultrastructure of the four cotton nectary types throughout development and compare these with the metabolomes of secreted nectars. Integration of these datasets supports specialization among nectary types to fulfill their ecological niche, while conserving parallel coordination of the merocrine-based and eccrine-based models of nectar biosynthesis. Nectary ultrastructures indicate an abundance of rough endoplasmic reticulum positioned parallel to the cell walls and a profusion of vesicles fusing to the plasma membranes, supporting the merocrine model of nectar biosynthesis. The eccrine-based model of nectar biosynthesis is supported by global transcriptomics data, which indicate a progression from starch biosynthesis to starch degradation and sucrose biosynthesis and secretion. Moreover, our nectary global transcriptomics data provide evidence for novel metabolic processes supporting de novo biosynthesis of amino acids secreted in trace quantities in nectars. Collectively, these data demonstrate the conservation of nectar-producing models among trichomatic and extrafloral nectaries.

## Teaser

Systems-based comparison of key metabolic modules in upland cotton (*Gossypium hirsutum*) nectaries indicates conservation of nectar-producing models in both trichomatic and extrafloral nectaries.

## Introduction

Domesticated Upland cotton, *Gossypium hirsutum*, develops a floral and three extrafloral nectaries, which are further subcategorized as vegetative or reproductive. All nectaries are trichomatic and secrete nectar from specialized papillae, a type of multicellular glandular trichome (Wergin et al., 1975). The secreted nectars are sugar-rich solutions presented as a reward to attract animal mutualists in exchange for ecosystem services (Simpson and Neff, 1981; Mitchell et al., 2009; Ollerton, 2017). The timing of nectar secretion varies among the different cotton nectaries, and is optimized for planta benefits, while minimizing the energetic cost of producing the nectar (Pleasants, 1983; Wäckers and Bonifay, 2004; Heil, 2011). Although cotton is largely a self-pollinating crop, honey bee visitations are facilitated by the floral nectar reward and increases boll and lint mass yield (Gilliam et al., 1981; Rhodes, 2002). However, the extrafloral nectars provide a source of indirect protection by attracting aggressive predatory ants, which ward off herbivores (Bentley, 1977; Wäckers et al., 2001; Rudgers et al., 2003; Rudgers and Strauss, 2004; González-Teuber et al., 2012). Consequently, the extrafloral nectaries modulate nectar secretion based on the environmental stress of insect herbivory (Wäckers and Bonifay, 2004). Overall, these plant–animal mutualisms improve plant fitness and reproductive success (Ollerton, 2017).

**Figure 1:**
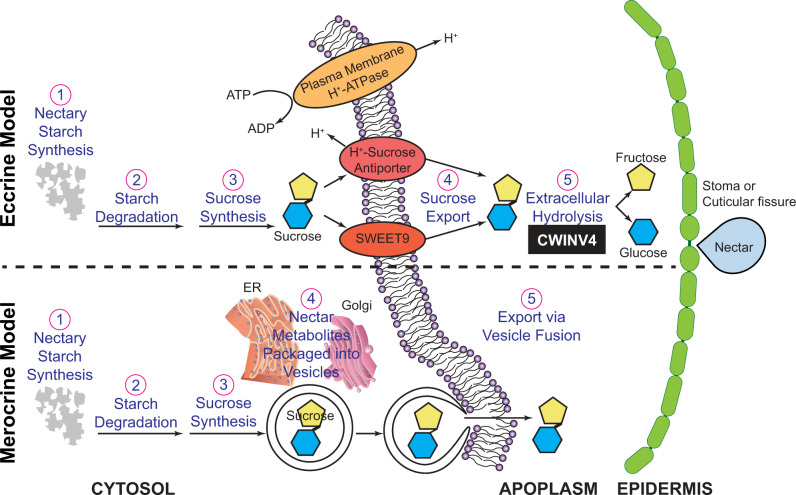
Comparative summary of the eccrine- and merocrine-based models of nectar synthesis and secretion. In the eccrine-based model, starch accumulates in the nectary tissue (1) and is subsequently degraded (2) to provide precursors for the biosynthesis of sucrose (3). Export of sucrose through the plasma membrane into the apoplasm (4) is facilitated by transmembrane transporters, such as the uniporter SWEET9 (Lin et al., 2014) or possibly by H^+^-sucrose antiporters (Vassilyev, 2010). Finally, sucrose in the apoplasm is hydrolyzed (5) by CELL WALL INVERTASE4 (Ruhlmann et al., 2010). In the merocrine-based model, processes 1–3 are synonymous to the eccrine-based model. Sucrose and other nectar metabolites are packaged into endoplasmic reticulum (ER) and/or Golgi-derived vesicle (4), which fuse with the plasma membrane (5) to release the vesicle content into the apoplasm.

The most prevalent models of nectar synthesis and secretion are the merocrine- and eccrine-based models summarized in Figure 1. The merocrine model is based primarily on ultrastructural analyses, which suggest that nectar metabolites are packed into vesicles derived from the endoplasmic reticulum or Golgi-derived vesicles, which fuse with the plasma membrane for secretion (Fahn, 1979; Heil, 2011; Roy et al., 2017). Studies using “omics” technologies on the floral nectaries of Arabidopsis, *Cucurbita pepo*, and *Nicotiana* spp. (Ren et al., 2007; Kram et al., 2009; Lin et al., 2014; Solhaug et al., 2019) have provided evidence that support an eccrine-based model. In this model, pores and transporters are used to move “pre-nectar” sugar metabolites through the plasma membrane of nectariferous parenchyma tissues (reviewed by Roy et al., 2017), and these sugars are transiently stored as starch in the nectary parenchyma (Peng et al., 2004; Ren et al., 2007; Lin et al., 2014; Chatt et al., 2018; Solhaug et al., 2019). At the time of nectar secretion, the stored starch is rapidly hydrolyzed and converted to sucrose and exported. Subsequent hydrolysis of sucrose by a cell wall invertase can generate glucose and fructose, thereby maintaining the sucrose concentration gradient needed for passive secretion (Ruhlmann et al., 2010; Lin et al., 2014). The last step of sucrose hydrolysis is also critical to the production of hexose-rich nectars (Ruhlmann et al., 2010), but this plays a minimal role in the production of sucrose-rich nectars (Chatt et al., 2018; Solhaug et al., 2019).

The release of nectar components from the nectary can be facilitated by the occurrence of modified stomata called “nectarostomata” (Decraene and Smets, 1991; Paiva, 2017). However, with trichomatic nectaries that do not have nectarostomata, nectar secretion involves passage through the cell wall and cuticle, but the mechanism is unclear. Based on ultrastructural analyses, at the time of nectar secretion, the cuticle appears to separate from the cell wall on the terminal cells of the glandular trichome, and nectar accumulates in the space between the cuticle and cell wall, thereby generating hydrostatic pressure that forces discrete nectar droplets through the porous cuticle (Findlay et al., 1971b; Wergin et al., 1975; Eleftheriou and Hall, 1983a; Kronestedt et al., 1986). It is unclear if there are biochemical alterations in the cell wall and cuticle to facilitate this process, or if the process is purely driven by a physical force that causes the cuticle to rupture.

In this study, a holistic system approach was applied to characterize the morphology, ultrastructure, and gene expression patterns of *G. hirsutum* floral and extrafloral nectaries as they develop through three developmental stages: (1) pre-secretory stage; (2) secretory stage; and (3) post-secretory stage. These comparisons were designed to identify signature morphological and biochemical alterations in the nectaries coincident with nectar secretion, and to assess whether the mechanisms of nectar secretion are common among the different nectary types. Specifically, the comprehensive datasets were compared with predictions made by the current merocrine- and eccrine-based models of nectar synthesis, to assess whether these models are conserved among trichomatic and extrafloral nectaries.

## Results

**Figure 2:**
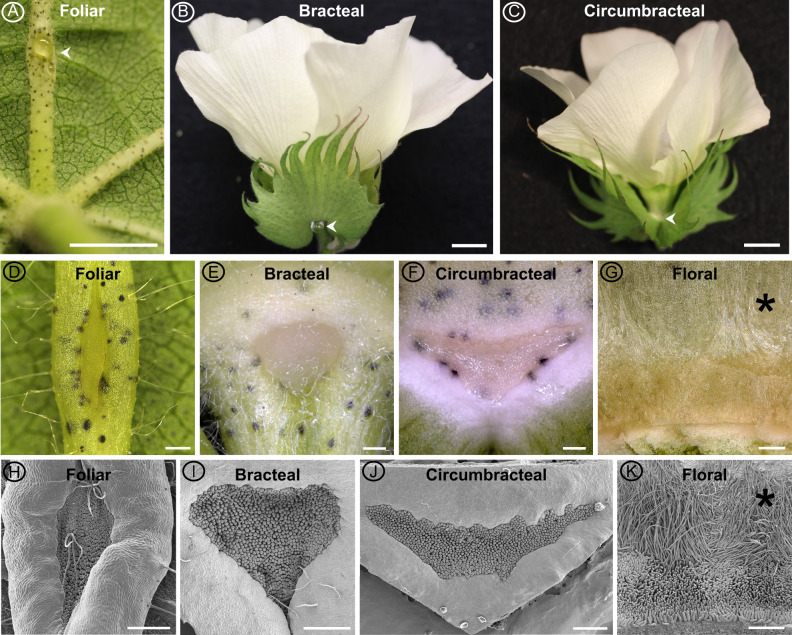
Macrostructure of different *G. hirsutum* nectaries at the secretory stage of development. Images obtained using a Canon EOS DSLR camera equipped with a 50-mm macro lens (A–C), macrozoom microscope (D–G), and SEM (H–K). Extrafloral nectaries are identified by arrow heads in panels A–C. The extrafloral nectaries (D–F and H–J) are composed of a pit of densely packed papillae. Floral nectary (G, K) is composed of a ring of stellate trichomes (*) subtended by a ring of papillae. Foliar nectary (A, D, and H); Bracteal nectary (B, E, and I); Circumbracteal nectary (C, F, and J); Floral nectary (G and K). Scale bar A = 5 mm; B and C = 10 mm; and D–K = 0.5 mm.

Domesticated Upland cotton, *G. hirsutum* (TM-1), develops four types of nectaries, three are extrafloral and one is floral, and all consist of multicellular glandular trichomes, specifically called papillae (Figure 2). The three extrafloral nectary types are subcategorized as vegetative (i.e., foliar nectary) or reproductive (i.e., bracteal and circumbracteal nectaries). The vegetative foliar nectary is located on the abaxial surface of the leaf midrib (Figure 2, A, D, and H). The reproductive bracteal and circumbracteal nectaries are associated with the flowers. The bracteal nectaries develop at the base of each bract subtending the flower and framing the cotton boll (Figure 2, B, E, and I). The circumbracteal nectary occurs on the abaxial calyx surface alternate with the bracts (Figure 2, C, F, and J). The floral nectary develops on the adaxial calyx surface and lines the basal circumference, with the secretory papillae subtending a ring of stellate trichomes (Figure 2, G and K).

### Nectar metabolome

In addition to their positional distinctiveness, metabolomic analysis of the nectars produced by each of the four cotton nectaries suggested functional differences. Each of these nectaries produce nectar at different rates, and in response to different developmental or environmental stimuli (Hanny and Elmore, 1974; Gilliam et al., 1981; Stone et al., 1985; Wäckers and Bonifay, 2004). Metabolite profiling experiments of nectar isolated from each nectary detected and quantified 197 analytes, with the successful chemical identification of 60 metabolites (Supplemental Dataset S1). The identified metabolites included the dominant sugars (sucrose, glucose, and fructose), as well as minor components, including amino acids, sugar alcohols, lipids, diols, organic acids, esters, and aromatics. Although there were quantitative differences in the components of the four nectars, each nectar contained a small number of metabolites that were unique to each nectary. Thus, there were 13, 5, 2, and 7 unique metabolites in the nectar produced by floral, bracteal, circumbracteal, and foliar nectaries, respectively.

The major constituents of the four nectars are glucose and fructose, which occur at an equal molar ratio, and account for more than 85% of the nectar sugars (Table 1). Variation between floral, reproductive extrafloral, and vegetative extrafloral nectars is clearly illustrated by the sucrose abundance, which accounts for 15% of the sugars in the foliar nectar, whereas in the other three nectars sucrose accounts for less than 6% of the nectar sugars (Table 1).

**Table 1:** Abundance of predominant sugars, and amino acids in different *G. hirsutum* nectars

Nectar type	Sugars (M)			Fructose-to-glucose ratio	Sucrose-to-hexose ratio	Amino acids (µM)			
	Fructose	Glucose	Sucrose			Essential	Non-essential	Non-proteinaceous	Total
Floral	1.81 ± 0.14^A^	1.89 ± 0.18^A^	0.005 ± 0.001^A^	0.97 ± 0.03	0.0014 ± 0.0004	116 ± 11	2950 ± 294	3.9 ± 1.2	3070 ± 303
Bracteal	4.05 ± 0.32^B^	4.27 ± 0.32^B^	0.50 ± 0.06^B^	0.95 ± 0.01	0.060 ± 0.005	12 ± 7	37 ± 12	5.7 ± 0.9	54 ± 20
Circumbracteal	4.3 ± 0.6^B^	4.3 ± 0.7^B^	0.37 ± 0.07^B^	1.02 ± 0.03	0.040 ± 0.004	1.9 ± 0.4	21 ± 3	6.8 ± 2.8	30 ± 2
Foliar	4.5 ± 0.4^B^	4.2 ± 0.3^B^	1.3 ± 0.1^C^	1.10 ± 0.01	0.150 ± 0.003	11 ± 6	40 ± 13	3 ± 1	55 ± 15

Different superscript letters indicate statistically significant differences in abundance based on an *F*-test followed by estimating the number of true null hypotheses using the jabes.q function described by Nettleton et al. (2006) (*q*-value < 0.05; *n* = 6).

**Figure 3:**
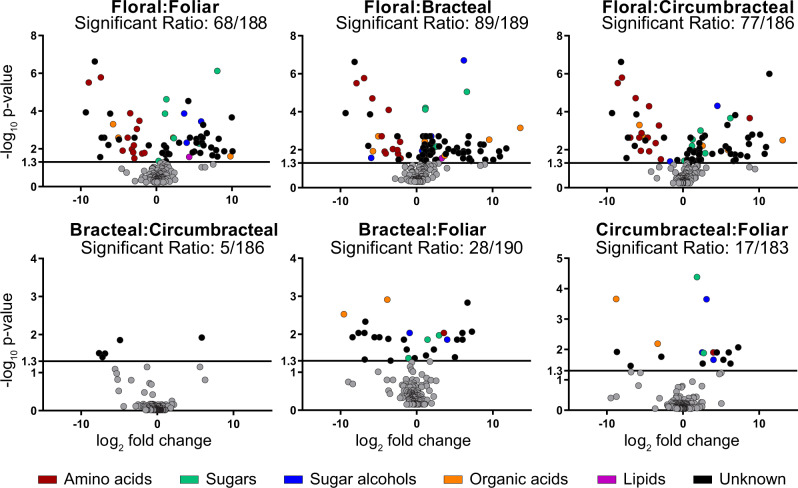
Volcano plot analyses of all possible pairwise comparisons of *G. hirsutum* nectar metabolomes. In each comparison, “Significant Ratio” identifies the proportion of the detected analytes whose abundance difference is statistically significant, as determined by an *F*-test; these data points are non-gray colored and are above the *y*-axis value of 1.3 (*q*-value < 0.05; *n* = 6). The chemical class identity of the metabolites is color-coded for those metabolites that show statistically significant abundance difference. Gray data points represent metabolites whose abundance is not statistically significant in the pairwise comparison.

Moreover, the four different nectar types can also be distinguished based on the minor nectar metabolites, particularly upon comparing floral and the extrafloral nectars (Supplemental Figures S1, S2). These compositional variations are visualized by the pairwise volcano plots shown in Figure 3, which reveal that 105 of the 197 detected analytes significantly differ in abundance in at least one pairwise comparison (*q*-value < 0.05, Supplemental File S1). The floral nectar is compositionally most distinct from the extrafloral nectars, with at least 68 distinguishing analytes between the former and each of the extrafloral nectars (Figure 3, A–C). Specifically, the amino acids are more abundant in the floral nectar (Table 1 and Supplemental Figure S2, Cluster 8), particularly aspartic acid, asparagine, leucine, phenylalanine, tryptophan, and γ-aminobutyric acid (GABA), which occur exclusively in the floral nectar (Supplemental Figure S1 and Supplemental Dataset S1). Another distinguishing compositional difference among the amino acids profiles is the high proportion of non-proteinaceous amino acids present in the extrafloral nectars, largely composed of β-alanine (i.e., 6%–20% in extrafloral nectar, compared with 0.05% of floral nectar; Table 1 and Supplemental Figure S3).

### Morphological features of nectary papillae

**Figure 4:**
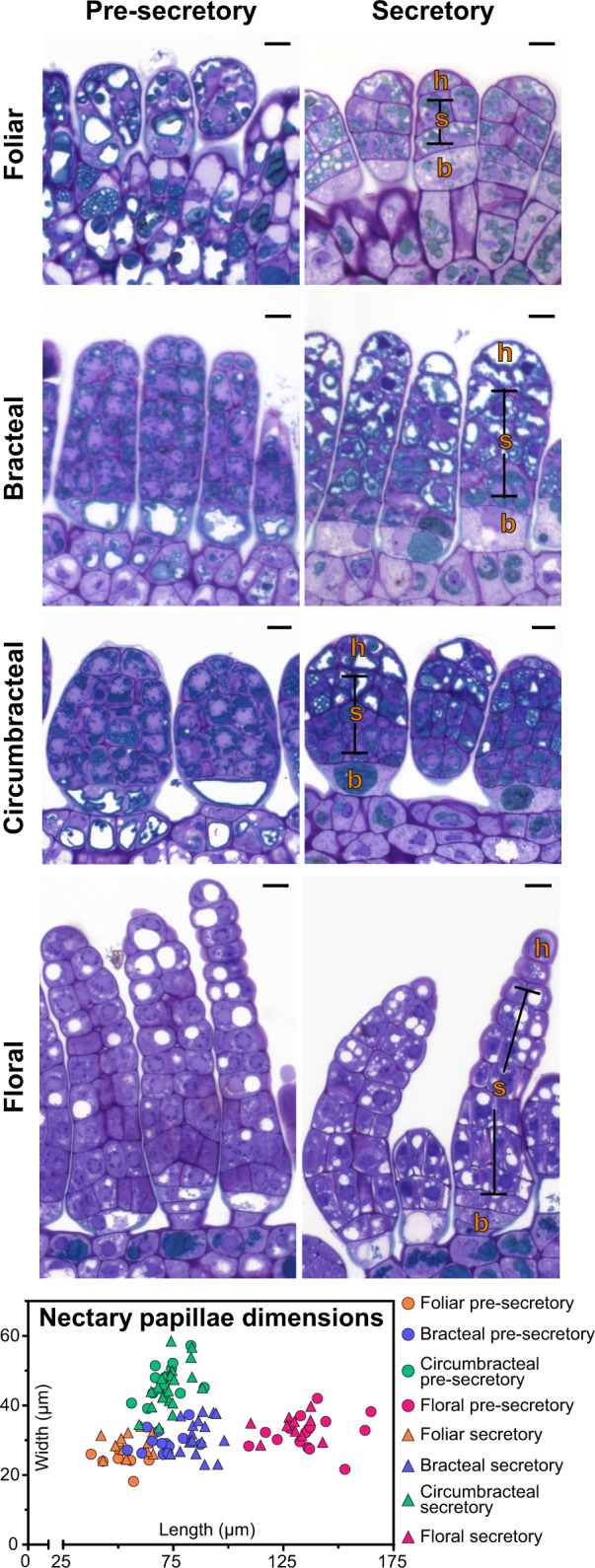
Light micrographs of longitudinal sections of papillae from the four different *G. hirsutum* nectary types, stained with toluidine blue O. The nectary papillae dimensions graph plots the length and width distribution of nectary papillae at different stages of development (*n* = 7–22 papillae for each nectary type). Abbreviations: h = head cells; s = stalk cells; b = basal cells. All scale bars = 10 µm.

The papillae of all four nectary types are multicellular and contain three regions typical of glandular trichomes; these being basal cell(s), stalk cells, and head cells. Extrafloral papillae at the secretory stage contain five to six layers of cells with an average papillae length of 68 ± 14 µm (sd), while the floral papillae are more extensive, with 12–14 cell layers with an average papillae length of 133 ± 10 µm (sd; Figure 4).

Regardless of papillae length, each papilla begins with distinct basal cell(s), which lack electron-dense cytoplasm. The three extrafloral nectaries contain a single basal cell (Figure 4), while the floral nectary contains two basal cells (Figure 4). The stalk cells, characterized by abundant phenolic bodies and vacuoles, determine the papillae length and width, and the circumbracteal nectaries have the widest papillae (46 ± 6 µm), when compared with the papillae of the other three nectaries (30 ± 4 µm; Figure 4). The densely staining bodies in the stalk cells of the bracteal and circumbracteal nectaries are arranged around the cell periphery (Figure 4).

The size and number of vacuoles differ among the cells of different types of nectaries and these attributes are also affected by nectary development. At the pre-secretory stage, stalk and head cells of bracteal and circumbracteal nectaries contain virtually no vacuoles (Figure 4), while at the secretory stage the distal two-thirds of the papillae cells become highly vacuolated, especially the head cells (Figure 4). In contrast to these nectaries, the stalk and head cells of pre-secretory stage foliar and floral nectaries contain large, circular vacuoles in cross-section (Figure 4), and by the secretory stage these vacuoles become smaller, and more numerous within the distally located cells of the papillae (Figure 4).

**Figure 5:**
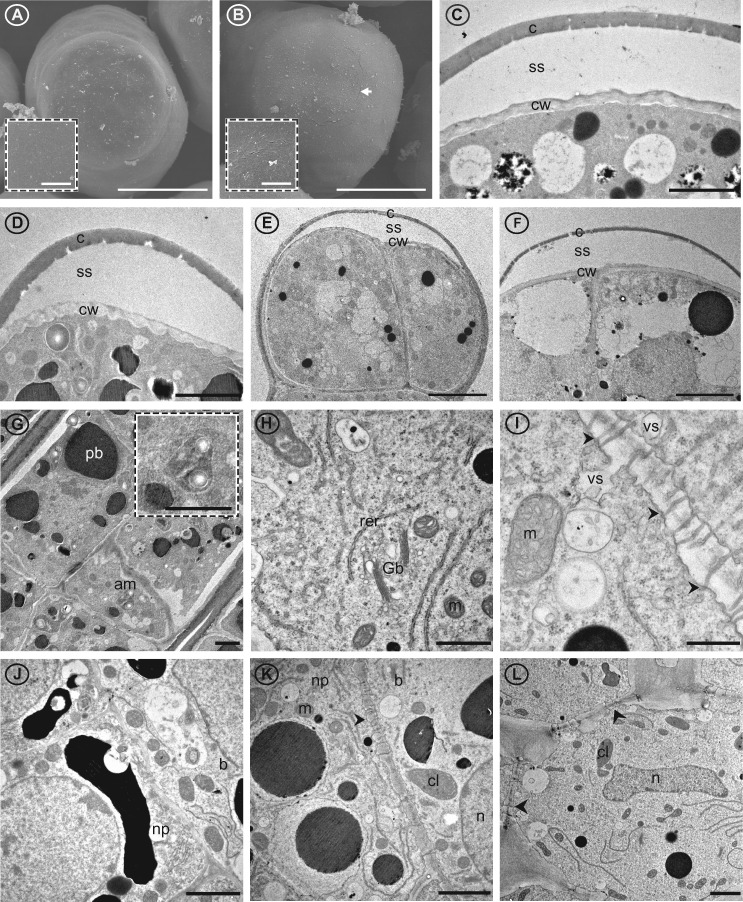
Cuticle, cell wall, and cellular details of *G. hirsutum* nectary papillae and supporting nectariferous parenchyma tissue. Images from scanning (A and B) and transmission (C–L) electron microsopy. A, Terminal end of papillae of circumbracteal nectary at pre-secretory stage, note lack of microchannels (cracks) in cuticle surface; B, Circumbracteal secretory nectary, arrow head identifies the microchannels in the cuticle surface; C–F, Head cells from secretory papillae showing separated cuticle of (C) circumbracteal, (D) bracteal, (E) floral, and (F) foliar nectaries; G, Stalk cells from bracteal nectary with amyloplast insert; H, Organelles of stalk cell exemplified by foliar nectary; I, Plasmodesmata (arrowheads) in cell wall of internal stalk cell; **J**, Basal cell from circumbracteal nectary; K, Junction between basal cell and nectariferous parenchyma of bracteal nectary; L, Nectariferous parenchyma from foliar nectary. Arrowheads identify plasmodesmata. Abbreviations: am = amyloplasts; c = cuticle; cl = chloroplast; cw = cell wall; b = basal cell; er = endoplasmic reticulum; Gb = Golgi body; m = mitochondria; n = nucleus; np = nectariferous parenchyma; pb = phenolic body; rer = rough endoplasmic reticulum; ss = subcuticular space; va = vacuole; vs = vesicle. Scale bars A and B = 25 µm; C, D, G, J, K, and L = 2 µm; E and F = 5 µm; H = 1 µm; I = 0.5 µm.

The cuticle and cell wall of the papillae have notable characteristics that are common among the four nectary types. Specifically, at the secretory stage, the cuticle of the head cells separates from the underlying cell wall and displays microchannels (Figure 5, A–F). These microchannels are visible as slits on the outer surface of the papillae head cells (Figure 5, B and C), but they are absent during the pre-secretory stage (Figure 5, A). In the case of the bracteal and circumbracteal nectaries, the separation of the cuticle from the cell wall occurs earlier in the development of the nectary, at the pre-secretory stage (Supplemental Figure S4, E). In addition, cell wall ingrowths toward the plasma membrane were observed in the bracteal and circumbracteal papillae head cells at the secretory stage (Figure 5, C and D).

### Organelle composition of nectary papillae

Transmission electron microscopy (TEM) examination of the papillae glands and supporting nectariferous parenchyma revealed the organelle composition of these cells (Figure 5 and summarized in Supplemental Table S1). All cells of the papillae of all four cotton nectaries are nucleated. The most common organelles observed in these cells are mitochondria, rough endoplasmic reticulum, and vesicles (Figure 5, H and I), whereas Golgi bodies (Figure 5, H) and amyloplasts (Figure 5, G) are significantly less abundant, and simple chloroplasts only occur in the bracteal (Figure 5, K) and foliar (Figure 5, L) nectaries. The basal cells of the papillae glands appear to have higher organelle complexity, containing more mitochondria and rough endoplasmic reticulum per cell (Figure 5, G–L), while the head cells display the least organelle complexity (Figure 5, C–F). Throughout the papillae and nectariferous parenchyma, vesicle fusing to the plasma membrane was frequently observed (Figure 5, I), and typical of nectary tissue, plasmodesmata traverse the inner anticlinal and peridermal walls of these tissues (Figure 5, I, K, and L).

### Morphological features of nectary parenchyma tissues (sub-papillae)

**Figure 6:**
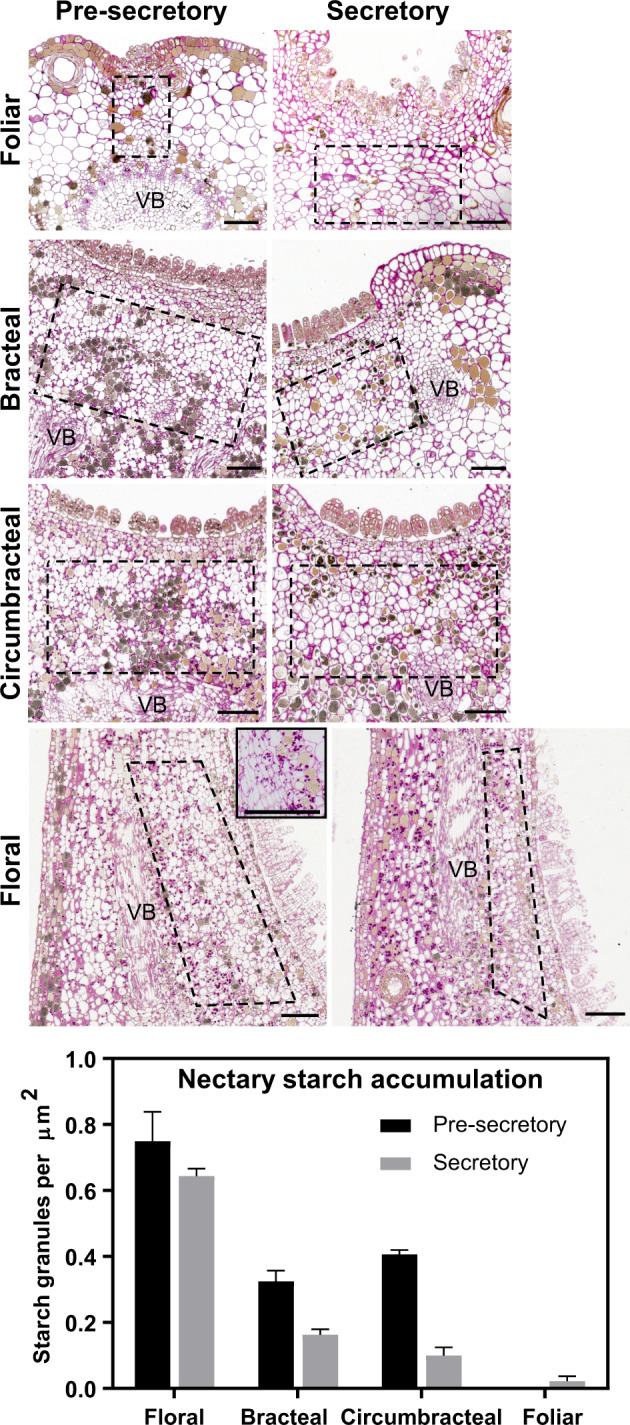
Light micrographs of longitudinal sections of different *G. hirsutum* nectaries stained with PAS stain to visualize distribution of starch granules within subnectariferous parenchyma (regions within dashed boxes) during nectary development. The nectary starch accumulation graph plots the density of starch granules within the subnectariferous parenchyma of nectaries during nectary development. For each nectary type and developmental stage, starch granules were counted from a minimum of six sections imaged from two separate nectaries. Error bars represent se. Abbreviations: VB = vascular bundle. All scale bars = 100 µm.

The nectariferous parenchyma of all nectary types is located between the subnectariferous parenchyma and the secretory papillae, and is characterized by isodiametric cells with minimal intercellular spaces. These cells display densely staining cytoplasm and contain diminutive phenolic bodies which are identified by heavy staining with toluidine blue O and osmium tetroxide (Supplemental Figure S4, A and D). The subnectariferous parenchyma is composed of approximately 10 layers of large cells, and the cytoplasmic density of these cells is less than that of the nectariferous parenchyma cells (Supplemental Figure S4, A and C). Vascular bundles are present near the subnectariferous parenchyma with phloem rays extending exclusively into the subnectariferous parenchyma of the foliar nectary (Supplemental Figure S4, B). The subnectariferous parenchyma of all examined nectary types form phenolic bodies as indicated by their golden-colored Periodic Acid Schiff’s (PAS) staining, and occur more abundantly in cells surrounding the vascular bundles (Figure 6). Similarly, cells containing druses (spherical aggregates of calcium oxalate crystals) were primarily observed surrounding the vascular bundles in the subnectariferous parenchyma of all nectary types. The druses present in foliar nectaries align in a row, in parallel to the phloem rays from the vascular bundles to the papillae (Figure 6).

Starch often serves as a source of nectar sugars (Ren et al., 2007). Starch accumulation within the subnectariferous parenchyma was visualized by fuchsia PAS staining at both the pre-secretory and secretory stages. In the floral, bracteal, and circumbracteal nectaries, starch granules form near the vascular bundles (Figure 6), and the frequency of these granules decreases as the nectaries transition from the pre-secretory to the secretory stages (Figure 6). In contrast, virtually no starch granules were observed within the subnectariferous parenchyma of the foliar nectaries at either developmental stages (Figure 6).

### RNA sequencing of cotton nectaries

Major changes in gene expression are associated with the transition from pre-secretory to secretory nectaries in several species (Solhaug et al., 2019). As such, the transcriptomes of the four cotton nectary types were resolved through three developmental stages, pre-secretory, secretory, and post-secretory (defined in the “Materials and methods” section). Using RNA-seq, over 360 M sequencing reads (125 bp, paired end) were generated from RNA isolated from the four cotton nectaries and from the adjacent non-nectary tissue; the latter was used to determine the transcriptomes of the non-nectary control tissues. These reads were initially mapped to the UTX-JGI *G. hirsutum* genome (v1.1) and subsequently mapped to *Arabidopsis thaliana* Col-0 genome. The latter was selected because the Arabidopsis genome is well annotated and has served as the genetic model for plant biology, including the process of nectar production (reviewed in Roy et al., 2017; Supplemental Dataset S2).

**Figure 7:**
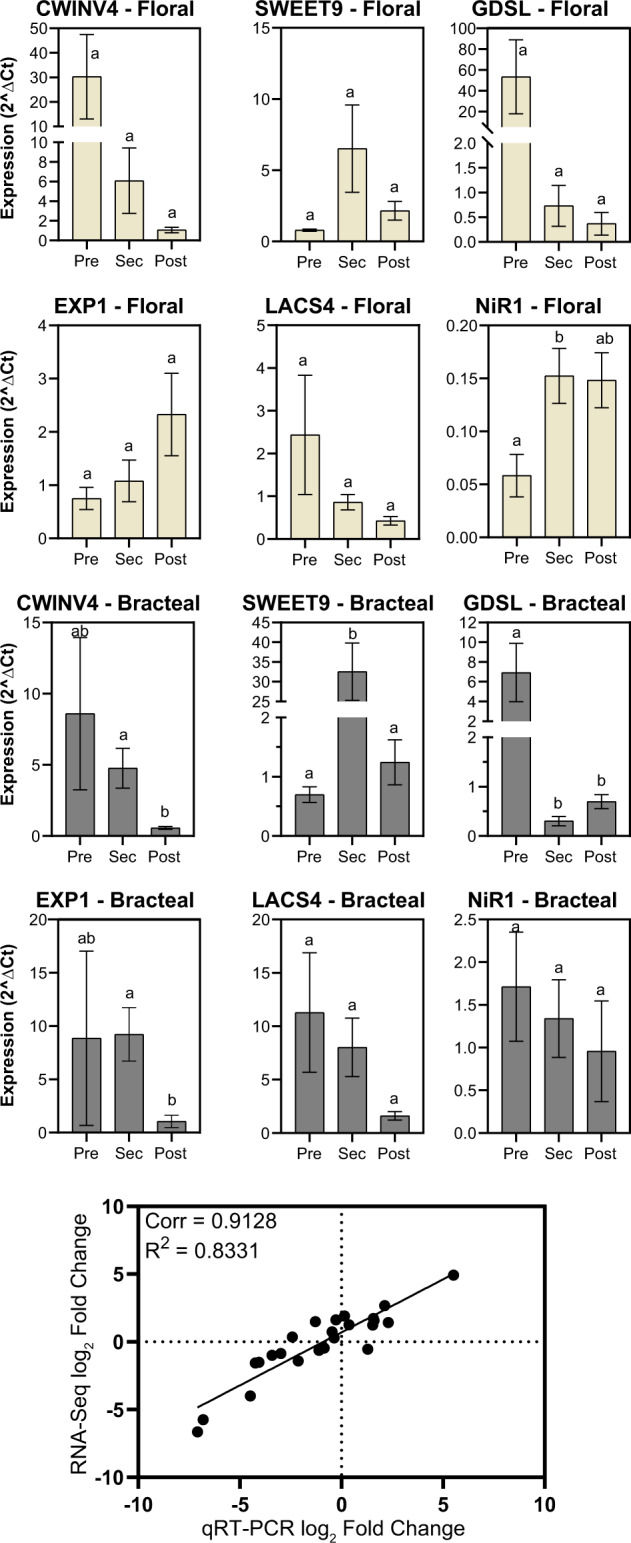
Validation of RNA‐seq data by RT-qPCR analysis. The identical RNA samples were subjected to parallel RNA‐seq analysis and RT-qPCR analysis. These latter analyses were targeted to the expression of six specific genes: *CWINV4* (*Cell Wall Invertase 4*), *EXP1* (*Expansin1*); *NiR1* (*Nitrite reductase 1*); *LCAS4* (*long chain acyl-CoA synthetase 4-like*); *GDSL* (*GDSL-like* Lipase/acylhydrolase); and *SWEET9* (*Sugars Will Eventually be Exported Transporter 9*). Expression was evaluated during the development of floral and bracteal nectaries as they transition from pre-secretory (Pre) to secretory (Sec) and to post-secretory (Post) stages, and the data are expressed as relative expression (2^ΔCt^), normalized to an internal control gene. Error bars represent standard error from a total of three biological replicates. Different letters above each data-bar indicates statistical significance in abundance (Student’s *t* test, *P*-value < 0.05). The scatter plot displays the Pearson’s correlation analysis between the RNA-seq and RT-qPCR datasets, expressed as fold-change in expression relative to the pre-secretory stage.

Expression profiles identified via RNA-seq analysis were validated by reverse-transcription quantitative PCR (RT-qPCR) analysis using the RNA isolated from floral and bracteal nectaries. These validation experiments targeted genes based on their known or suspected functionality in nectary development (Kram et al., 2008; Ruhlmann et al., 2010; Lin et al., 2014; Solhaug et al., 2019). Some of the selected genes display distinctive differential expression during nectary development, while others show a more stable expression pattern (e.g., nitrite reductase 1, *NiR1*). The RT-qPCR expression data for the six selected genes were compared with the RNA-Seq expression values of these genes obtained from the floral and bracteal nectaries from different developmental stages. Pearson’s correlation analysis of these two datasets leads to the finding of a strong positive correlation between these two methods for measuring gene expression (*R*^2^ = 0.83; Figure 7). This strong correlation indicates, therefore, that the RNA-seq analyses can be used to draw conclusions concerning gene expression activity in developing nectary tissues.

The collected RNA-Seq data were analyzed using the DESeq statistical package (Anders and Huber, 2010), which initially identified differentially expressed genes (either up-regulated or down-regulated) between each nectary type and each adjacent non-nectary control tissue. Subsequent analyses focused on the genes that showed altered expression in the nectary samples, and these were evaluated to determine the effect of nectary development on the expression level. These analyses identified genes that show distinct differential expression patterns as each nectary type undergoes a different developmental trajectory, and those that share a common developmental expression pattern among all four nectaries, irrespective of the type of nectary (Supplemental Datasets S3, S4).

### Differential gene expression analyses

**Figure 8:**
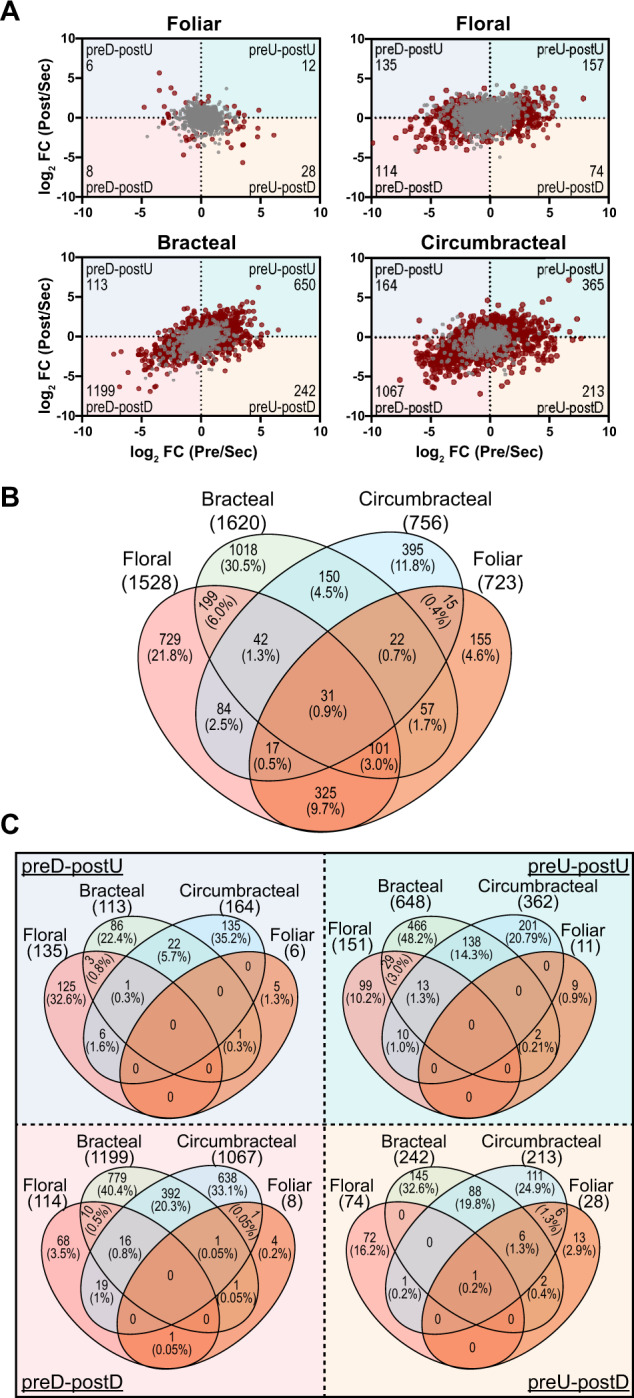
Differentially expressed genes in four nectary types. A, Scatter plots displaying differentially expressed genes in response to nectary development from presecretory (Pre) to secretory (Sec) to post-secretory (Post) stages, normalized relative to the expression level at the secretory stage. Gray colored data points represent genes that are preferentially expressed in each nectary type with respect to the adjacent non-nectary tissue, but expression is minimally affected by nectary development. Red colored data points represent genes that are differentially expressed in each nectary type, and expression is also modulated by the development of each nectary type. These red data points are divided into four quadrants, which detail changes in gene expression patterns normalized to the secretory developmental stage: (1) down-regulated at the pre-secretory stage and up-regulated at the post-secretory stage (preD–postU); (2) up-regulated at the pre-secretory stage and up-regulated at the post-secretory stage (preU–postU); (3) up-regulated at the pre-secretory stage and down-regulated at the post-secretory stage (preU–postD); and (4) down-regulated at the pre-secretory stage and down-regulated at the post-secretory stage (preD–postD). The number of differentially expressed genes in each sector is identified in the outer corner of each sector. B, Venn diagram representation of the distribution of genes that are preferentially expressed in each nectary type, but expression is not modulated by nectary development (i.e. the genes identified by gray data-points in panel A). The digits identify the absolute number and percentage of genes falling into each subset category. C, Venn diagram representation of the distribution of genes that show nectary-specific expression that also demonstrate temporal patterns of gene expression as the nectaries transition from presecretory, to secretory and to post-secretory stages of development (i.e. overlap among the genes represented by red-colored data-points in panel A.) The digits identify the absolute number and percentage of genes falling into each subset category.

The scatter plots shown in Figure 8, A (from Supplemental Dataset S3) summarize changes in gene expression for each of the four nectary types, as each nectary developmentally transitions from pre-secretory to secretory stages and from secretory to post-secretory stages. The gray data-points in these graphs identify the 3,337 genes whose expression is not temporally affected by the developmental trajectory of each nectary, but does differ when compared with the adjacent non-nectary control tissue. In addition, there are 3,434 genes that show a temporal change in expression as the nectaries transition between the three developmental stages (red datapoints in Figure 8, A and Supplemental Dataset S6).

**Figure 9:**
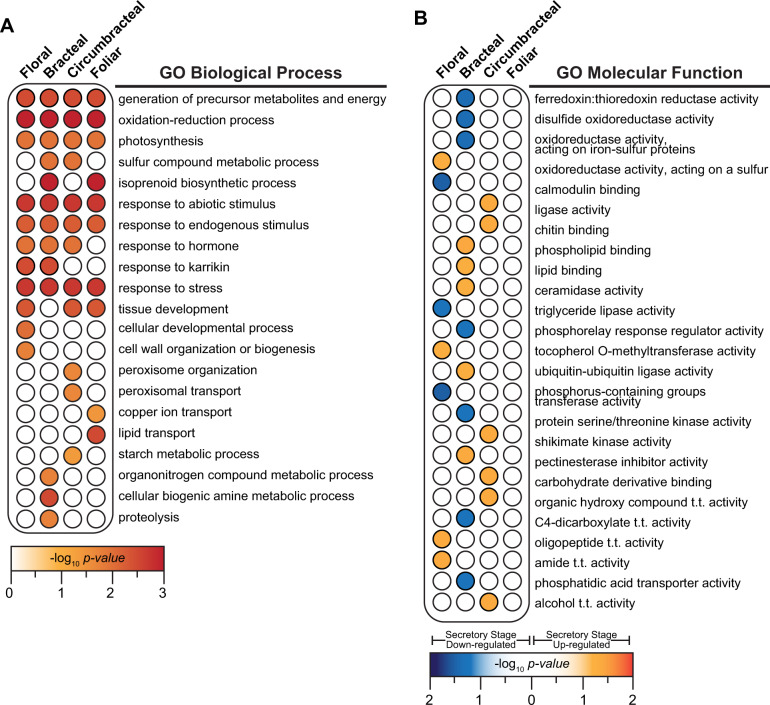
GO enrichment analysis. A, Genes identified in Figure 7, B that are preferentially expressed in each nectary type, but expression is not modulated by nectary development. B, Genes identified in Figure 7, C, as uniquely up- or down-regulated during the secretory stage of development in each nectary type. Redundancy in the lists of enriched GO terms was removed using REVIGO (Supek et al., 2011). Details of the results are provided in Supplemental Datasets S5, S8. Abbreviations: t.t. = transmembrane transporter.

The distribution of the genes whose expression is not affected by development (i.e., 3,337 genes) among the four different nectary types is visualized in the Venn diagram shown in Figure 8, B (Supplemental Dataset S4). Very few of these genes (<1%) are shared among all four of the nectary types, and only 31% of these genes are commonly shared among any 2 or more of the four nectary types. Gene ontology (GO) enrichment analyses of the genes not affected by development was completed for each of the four nectary types (i.e., 1,528 genes floral, 1,620 genes bracteal, 756 genes circumbracteal, and 723 genes foliar). This analysis identified molecular functions and biological process terminologies related to oxidoreductase catalytic activities and the generation of precursor metabolites and energy metabolism (Figure 9, A andSupplemental Dataset S5). These functionalities are consistent with the need to generate nectar precursor metabolites and cellular energetics, and we therefore surmise these are basal processes that are commonly required in maintaining an operational nectary, independent of the nectary type and independent of the developmental trajectory of the nectary. However, a significant proportion of these genes are uniquely expressed in each nectary type: 22% in floral nectaries, 30% in bracteal nectaries, 12% in circumbracteal nectaries, and 5% in foliar nectaries. These nectary specific subset of genes whose expression is unaffected by nectary development identify biological processes that are distinct for each nectary type and thus highlight metabolic variation among the nectaries. These include for example isoprenoid biosynthesis, metabolism of sulfurous compounds, lipid transport, and metabolism associated with amines and organonitrogen compounds (Figure 9, A).

The red data-points in each scatter plot of Figure 8, A are divided into quadrants detailing the four temporal patterns of differential gene expression associated with development of each nectary type; namely 3,434 genes show a temporal change in expression in at least one of these nectary developmental transitions. Each of these quadrants identify different temporal pattern of gene expression for each nectary type, namely genes that are: (1) down-regulated at the pre-secretory stage and up-regulated at the post-secretory stage (preD–postU quadrant); (2) up-regulated at the pre-secretory stage and up-regulated at the post-secretory stage (preU–postU quadrant); (3) up-regulated at the pre-secretory stage and down-regulated at the post-secretory stage (preU–postD quadrant); and (4) down-regulated at the pre-secretory stage and down-regulated at the post-secretory stage (preD–postD quadrant; Supplemental Dataset S6). The Venn diagrams in Figure 8, C (Supplemental Dataset S7) identify the number of genes that share these four temporal patterns of gene expression among the four nectary types.

These comparisons indicate that each nectary type displays a distinct temporal program of gene expression as they develop. Indeed, there is only a single gene, terpene synthase 21 (AT5G23960.2), which shares the same temporal expression pattern across all four nectary types. Analogously, the bracteal and circumbracteal nectaries display temporal gene expression profiles that are most similar to each other (sharing 17% of the differentially expressed genes), while the floral and vegetative foliar nectaries are the most distinct (sharing only 0.05% of the differentially expressed genes). Another feature that distinguishes the foliar nectaries is the sparcity of ∼50 genes whose expression is affected by the development of this nectary type (Figure 8, C). This is probably associated with the fact that nectar secretion by foliar nectaries occurs at a steady rate and low volume. In contrast, in the other three nectary types, between 470 and 1,800 genes uniquely modulate their expression level as each nectary undergoes development (Figure 8, C).

Enrichment of GO terms associated with each nectary-specific subset of genes provided insights on the functionalities that are modulated during the development of each nectary type (Supplemental Dataset S8). These broad cellular component, biological process, and molecular function categories define functional similarities and differences among the four nectary types. Specifically, Figure 9, B comparatively summarizes the developmentally modulated molecular functions between the four nectary types. These analyses indicate that each nectary expresses a genetic network that supports different biological processes. Thus, even though these four nectary types share a common morphology (i.e. papillae nectaries) and functionality (i.e. the production and secretion of nectar), different genetic networks appear to support these common morphological and functional features. For example, up-regulated genes of the floral nectary are enriched for “amide transmembrane transporter activity” (Figure 9, B), while down-regulated genes are enriched for processes related to lipid catabolism (Supplemental Figure S5). In contrast, the circumbracteal nectaries display up-regulation of fatty acid catabolism. Lastly, the bracteal nectary at the secretory stage displays an up-regulation of detoxification processes and responses to osmotic stress, potentially related to the concurrent up-regulation of autophagy and proteosomal protein degradation. There is also extensive up-regulation of cellular organization related to protein complexes, cellulose and pectin of the cell wall, and organelle membranes.

### Expression of carbohydrate metabolism genes related to nectar production

**Figure 10:**
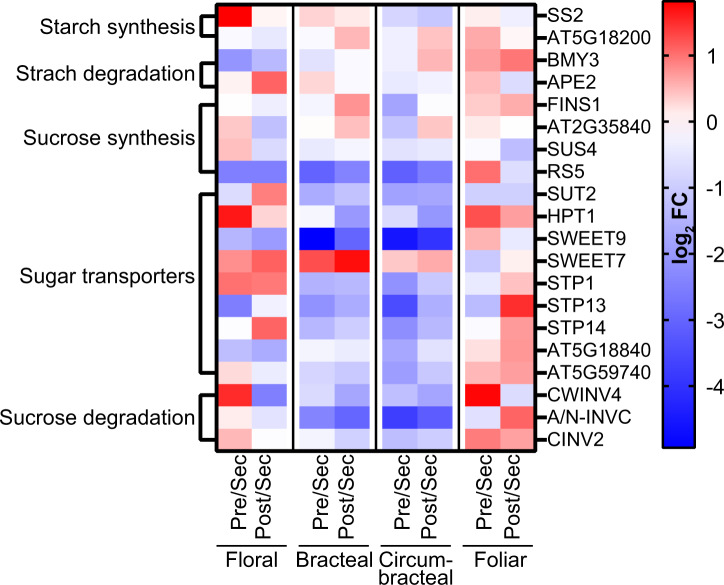
Expression analysis of genes involved in starch and sucrose metabolism. Normalized RNA-seq data were used to generate heat maps of changes in gene expression as each nectary-type transition from pre-secretory to secretory and from secretory to post-secretory stages of development. The blue–red color scale indicates the relative fold-change (FC) between these developmental transitions. Blue indicates increased expression at the secretary stage of nectary development and red indicates decreased expression at the secretary stage of nectary development. Full names for the abbreviations of individual genes are provided in Supplemental Dataset S9. Abbreviations: Pre = pre-secretory; Sec = secretory; and Post = post-secretory.

Because the primary metabolic process of nectaries is the production of sugar-rich nectar (Table 1), the RNA-seq data were mined for genes associated with sugar metabolism, including starch metabolism (Ren et al., 2007; Solhaug et al., 2019), and transmembrane transporters. The resulting gene list was further filtered for genes that are upregulated in the nectary transcriptomes relative to the adjoining control non-nectary transcriptomes. The heat map presented in Figure 9 illustrates the temporal expression patterns of the 20 selected genes among the four different nectary types as they each undergo development (Supplemental Dataset S10). Figure 10 displays these genes in sequential order of their functionality as predicted by the eccrine-based model of nectar secretion (reviewed by Roy et al., 2017).

In floral nectaries, these genes follow a developmental expression pattern that is most consistent with the carbohydrate metabolic events that are predicted by the eccrine model of nectar production; namely initial synthesis of starch at the presecretory stage, followed by conversion to nectar constituents, sucrose and hexoses, as the nectary develops to the secretory stages. Specifically, the upregulation of *SS2* (*Starch Synthase 2*) at the pre-secretory stage, followed by the higher expression of *BMY3* (*β-Amylase 3*), *SUS4* (*Sucrose Synthase 4*), *SWEET9*, and *CWINV4* (*Cell Wall Invertase 4*), which are genes associated with the conversion of starch to secreted sucrose and monosaccharides that occurs during the secretory stage of nectary development, consistent with this expectation.

In the bracteal and circumbracteal nectaries, the expression profiles of these genes deviate from the floral nectary profile, and thereby are less consistent with the eccrine model of nectar development. Nevertheless, in these two nectary types, the expression patterns that reflect the expectation of the eccrine model include those of *SUS4* and *RS5* involved in sucrose synthesis, the sugar:proton symporters, *SUT2*, *STP1*, *STP13*, and *STP14*, and a UDP-galactose antiporter (AT5G59740), which are highly expressed during the secretory stage. Thus, these sugar transporters, in addition to *SWEET9*, may contribute to nectar-sugar secretion in these two nectary types.

Finally, in the foliar nectary, the expression patterns of the carbohydrate metabolism and transporter genes align poorly with the expectation of the eccrine-based model of nectar production. However, this lack of a developmental correlation with the eccrine model may be associated with the fact that the foliar nectary constantly produces nectar, and thus expression of carbohydrate metabolism genes do not necessarily respond to developmental cues.

### Expression of transmembrane transporter genes related to nectar production

The transcriptomes of the four cotton nectaries are also enriched for transmembrane transporter genes whose expression is developmentally modulated. As would be expected for secretory organs, the expression of these transporter-coding genes generally peak during the secretory stage of nectary development (27% of foliar, 39% of floral, 81% of bracteal, and 86% of circumbracteal; Supplemental Figure S6). The 79 transporters identified are predicted to transport sugars, amino acids, water, and various ions (borate, phosphate, hydrogen, calcium, chloride, iron, potassium, and zinc; Supplemental Dataset S9). Transporters that commonly show peak expression at either the pre-secretory or secretory stage of all four nectary types include those needed for the movement of water via plasma membrane intrinsic proteins (AT2G37170, AT3G53420, AT2G45960 homologs). In contrast, the amino acid transporters, *PROT1* (AT2G39890), AT1G47670, and AT3G56200 homologs, show differential expression during the development of floral, bracteal, and circumbracteal nectaries, but not in the foliar nectary (Supplemental Dataset S9 and Supplemental Figure S6).

### Upregulation of nitrogen assimilation and amino acid biosynthesis within nectaries

**Figure 11:**
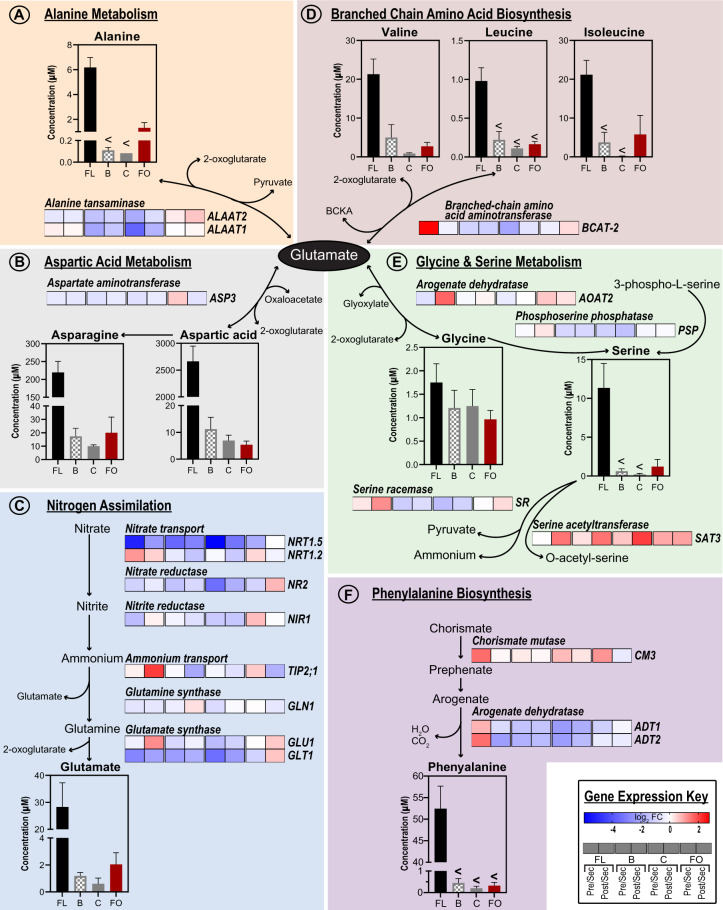
Integration of metabolomics and transcriptomics data to decipher the metabolic processes that support nitrogen assimilation and amino acid biosynthesis in nectaries. Each metabolic module (A–F) integrates gene expression data of enzymes catalyzing key metabolic processes leading to the production of metabolites detected in the nectar metabolome. The “Gene Expression Key” indicates the change in gene expression, as each nectary types develops from pre-secretory, secretory, and post-secretory. For each nectary type, change in gene expression is calculated as a ratio relative to the abundance of each transcript in the secretory stage of development. Gene descriptions are provided in Supplemental Dataset S9. Data-bars labeled with the “<” symbol indicate metabolites that accumulate at levels that are below the detection limit of the analytical method. Replication of the metabolomics data (*n* = 6) and transcriptomics data (*n* = 3) was enabled by the analysis of multiple biological samples. Abbreviations: FL = floral; B = bracteal; C = circumbracteal; FO = foliar; Pre = pre-secretory; Sec = secretory; Post = post-secretory.

The transcriptome data indicate that during the secretory stage of development, the floral, bracteal, and circumbracteal nectaries display upregulated expression of genes associated with nitrogen assimilation and metabolism. Specifically, the transcriptome profiles suggest that nitrate is initially transported through the xylem to the subnectariferous parenchyma by the cotton orthologs of the proton-coupled nitrate transporter *NRT1.5* (AT1G69850; Tsay et al., 2007; Lin et al., 2008), which displays peak expression during the secretory stage in the floral and reproductive extrafloral nectaries. The transcriptome data also indicate that once in the subnectariferous parenchyma, nitrate would undergo two successive reductions to produce ammonium (catalyzed by nitrate reductase *NR2*, AT1G37130 and nitrite reductase *NIR1*, AT2G15620), which could be used to assemble glutamine and glutamate (catalyzed by glutamine synthase [*GLN1*, AT5G37600] and glutamate synthase [*GLU1*]; reviewed by Bernard and Habash, 2009). Ammonium flux may be modulated by the tonoplast localized ammonium uniporter *TIP2;1* (Loque et al., 2005), a gene upregulated in secretory floral and reproductive extrafloral nectaries (Figure 11, C andSupplemental Dataset S9). The temporal expression pattern of these genes is distinct in the foliar nectaries, with very few being modulated by the development, which, unlike the other nectary types, constantly secretes nectar.

Because amino acids are the second most abundant class of nectar metabolites, we used MapMan (Thimm et al., 2004) and AraCyc (Mueller et al., 2003) to examine the nectary transcriptomes for genes associated with amino acid biosynthesis. We identified that the nectary transcriptomes show high expression of a set of genes that use glutamate as a substrate for the biosynthesis of other amino acids (Figure 11, A, B, D, and E). Specifically, these gene products are primarily involved in the biosynthesis of alanine, aspartate, glycine, and branched chain amino acids, and they show peak expression during the secretory stage of the floral, bracteal, and circumbracteal nectaries. An example of one of these glutamate utilizing enzymes is *aspartate aminotransferase 3* (*ASP3*, AT5G11520), which displayed the highest expression among all nectaries at the pre-secretory and secretory stages (Figure 11, B). This enzyme utilizes glutamate and oxaloacetate to produce aspartic acid, one of the most abundant amino acids of floral and extrafloral nectars. Other such correlations between nectar amino acids and biosynthetic enzymes include phenylalanine and the biosynthetic enzyme, arogenate dehydratase 2 (*ADT2*, AT3G07630; Figure 11, F), and proline and the proline transporter *PROT1* (AT2G39890, Supplemental Dataset S9 and Supplemental Figure S6; Yamada et al., 2011).

### Cell wall and lipid metabolism during nectar secretion

As indicated by the morphological studies of the nectary papillae, we anticipated that genes associated with cell wall and cuticle deposition may show altered expression during nectary development. Such genes were selected based on the spatial and temporal differential expression patterns as revealed by the RNA-seq data, and they were mapped to metabolic networks using MapMan (Thimm et al., 2004). Consistent with the morphological-based expectations, these analyses indicate that during bracteal and circumbracteal nectary development, cell wall and cuticle associated genes display temporal differential expression that peaks during nectar secretion (Supplemental Figures S7, S8 and Supplemental Dataset S9). Specifically, in these nectaries eight genes related to cell wall re-structuring are significantly upregulated during the secretory stage; these include an expansin (*EXLA1*, AT3G45970), and genes required for the synthesis of cell wall components such as callose (*GSL10*, AT3G07160), hemicellulose (*GALT6*, AT5G62620), and pectins (*PME17*, AT2G45220). Likewise, 17 genes related to cuticular lipid metabolism, including fatty acyl elongation, transport of lipids, including the transport of cutin precursors (*ABCG11*, AT1G17840) are commonly upregulated in these two nectary-types.

## Discussion

This study presents a system-based comparison of the four nectary types of *G. hirsutum*, expanding on earlier characterizations of the morphology and ultrastructure of the cotton foliar nectaries (Wergin et al., 1975; Eleftheriou and Hall, 1983a). Specifically, we compared and contrasted the morphologies and transcriptomes of the floral, bracteal, circumbracteal, and foliar nectaries, as well as their associated nectar metabolomes. These data build upon molecular models for nectar production developed primarily using floral nectaries of Arabidopsis and *Nicotiana* spp. (Carter et al., 1999, 2006, 2007; Carter and Thornburg, 2000, 2004; Thornburg et al., 2003; Ren et al., 2007; Kram and Carter, 2009; Hampton et al., 2010; Ruhlmann et al., 2010; Bender et al., 2012, 2013; Liu and Thornburg, 2012; Lin et al., 2014; Wiesen et al., 2016; Thomas et al., 2017), which secrete nectars through modified stomata, referred to as “nectarostomata.” Thus, this study evaluates the applicability of the nectar production models developed with nectarostomata-secreting nectaries to nectaries composed of secretory trichomes (papillae).

The study revealed metabolic processes that are temporally regulated as the papillae nectaries progress from the pre-secretory stage to secretory stage and to post-secretory stages of development. Regulation of these metabolic processes differs among the four cotton nectary types, potentially to fullfil the ecological niche that each nectary serves. Each of these four nectary types have distinct patterns of nectar secretion, with the floral and extrafloral nectaries (i.e., bracteal and circumbracteal nectaries) following ontogenetic patterns of secretion, while the foliar nectary displays low constitutive secretion, which can be upregulated upon herbivory (Wäckers and Bonifay, 2004). In line with these distinct patterns of nectar secretion are the equally distinct gene expression networks among the four nectary types with very few genes (<1%) sharing common expression patterns throughout development. Additionally, GO enrichment analysis using subsets of genes differentially expressed in a single nectary type at the secretory stage illustrated that each nectary carries out unique metabolic processes to facilitate nectar generation. For example, the bracteal nectary alone displayed an upregulation of genes associated with autophagy, a process previously implicated in the development of extrafloral nectaries (Machado and Rodrigues, 2019). It is possible that the bracteal nectary uses autophagy as a catabolic process to maintain precursor pools for nectar generation to sustain extended nectar secretion throughout cotton boll maturation (Wäckers and Bonifay, 2004; Marshall and Vierstra, 2018).

### Mechanisms of cotton nectar secretion supported by expression profiles and papillae ultrastructure

Figure 1 compares the merocrine and the eccrine models that have been proposed as potential mechanisms for the generation of nectar components and release from trichomatic nectaries (Findlay et al., 1971b; Kronestedt et al., 1986; Paiva, 2016). These mechanisms must explain how the nectar components cross the barriers posed by the plasma membrane, cell wall, and cuticle. The potential complexity of this process is multiplied considering the variation between floral and extrafloral nectaries which have contrasting patterns of nectar secretion and the metabolic origins of precursors for nectar metabolites (e.g., starch as precursors of sugar components). Ultrastructural and transcriptome characterizations of the cotton nectaries support the cooccurrence of both the merocrine and the eccrine model of nectar generation. These two models likely function in coordination with each other to synthesize nectar components and secrete the metabolites from the nectary tissues. In the merocrine model, nectar metabolites are packaged into vesicles that fuse with the plasma membrane, releasing the nectar components.

The role of the merocrine-based secretion in cotton nectaries is best supported when one considers the ultrastructural analyses which illustrate shared structural components among the four nectary types of cotton, similar to the trichomatic nectaries reported in other taxa, such as *Abutilon* (Kronestedt et al., 1986), *Hibiscus* (Sawidis et al., 1987), *Platanthera* (Stpiczyńska et al., 2005), and *Utricularia* (Plachno et al., 2018). Specifically, the prominence of rough endoplasmic reticulum positioned parallel to the cell walls may contribute to vesicle trafficking (Eleftheriou and Hall, 1983a), and there is an abundance of such vesicles fusing to plasma membranes within the nectariferous parenchyma and throughout the papillae of cotton nectaries. These features are consistent with the importance of vesicle movement to deliver and secrete nectar components in the merocrine model.

The eccrine model deviates from the merocrine, in that nectar metabolites are ferried through the plasma membrane by channels and transporters (reviewed by Roy et al., 2017). In both models, prior to the final release of nectar by vesicles or transporters, the pre-nectar metabolites move symplastically through the nectar parenchyma tissues (Findlay et al., 1971a; Wergin et al., 1975; Eleftheriou and Hall, 1983a). This symplastic flow of pre-nectar metabolites is supported in this study by the abundance of plasmodesmata pit fields traversing the cell walls of the nectariferous parenchyma and the inner anticlinal and peridermal walls of the papillae.

Furthermore, the transcriptome expression patterns during the development of floral and reproductive extrafloral nectaries support the eccrine model. Based on molecular evidence from floral nectaries of Cucurbitaceae, Brassicaceae, and Solanaceae (Lin et al., 2014; Ruhlmann et al., 2010; Thomas et al., 2017; Chatt et al., 2018; Solhaug et al., 2019), the eccrine model is composed of at least five metabolic processes: (1) starch synthesis, (2) starch degradation, (3) sucrose synthesis, (4) export of sucrose into the apoplast via SWEET9, and (5) extracellular hydrolysis of sucrose by CELL WALL INVERTASE4 (CWINV4). The anticipated expression of genes coding for enzymes and transporters associated with these five metabolic processes was conserved among the floral and reproductive extrafloral nectaries of cotton (Figure 7 and Supplemental Dataset S2), which was also supported by starch accumulation patterns (Figure 6). The lack of such an expression pattern during the development of foliar nectaries may be a consequence of the fact that these nectaries produce a steady but low volume of nectar, and thus there is no need for a change in a gene expression program.

The eccrine model of nectar deposition has been primarily developed to explain the deposition of the sugar components of nectars. Similarly, however, the expression of genes encoding transporters of the minor components of the nectars would indicate that the eccrine model applies equally to these classes of metabolites. In support of this hypothesis, the transcriptomes of developing cotton nectaries reveal upregulated expression of plasma membrane-H^+^-ATPase, sugar:proton symporters, amino acid transporters, and lipid transmembrane transporters at the secretory stage of nectary development. The expression of such ATPase transmembrane transporters and proton gradients has previously been suggested to facilitate export of nectar metabolites (Eleftheriou and Hall, 1983b; Peng et al., 2004; Bernardello et al., 2007; Vassilyev, 2010; Chatt et al., 2018). Moreover, the occurrence of calcium oxalate crystals (druses) around the vasculature and throughout the nectary parenchyma tissues may indicate the need to regulate calcium levels by sequestration as insoluble salts to negate the inhibitory effects of this cation on plasma membrane ATPases (Kronestedt et al., 1986; Aguero et al., 2018; Tölke et al., 2018).

### Distinct nectar compositions reflect the feeding preferences of target facultative mutualists that visit each nectary type

Our metabolic profiling of the cotton nectars extensively details the variation between the floral and extrafloral nectar compositions. The distinct nectars produced by cotton floral and extrafloral nectaries parallel the feeding preferences of the mutualists that visit these nectaries. The floral nectary is primarily visited by honey bee pollinators (Butler et al., 1972), while the extrafloral nectaries are visited by protective predatory ants (Hanny and Elmore, 1974). Specifically, reflecting that bees prefer to feed on hexose-rich nectar (Waller, 1972; Baker and Baker, 1983), the floral nectar is the most hexose rich of the nectars that are produced by the four nectary types, containing minimal amounts of sucrose. Similarly, multiple positive attributes have been associated between dietary amino acids and bee health, which include (1) the phagostimulatory effect of phenylalanine and GABA (Petanidou et al., 2006; Hendriksma et al., 2014; Nepi, 2014); (2) conferring health benefits by GABA-enriched artificial nectar (Bogo et al., 2019); (3) flavor stimulatory effects of leucine and tryptophan on sugar chemosensory cells (Shiraishi and Kuwabara, 1970); and (4) access to a rapid energy source for initial flight take-off provided by proline (Carter et al., 2006; Teulier et al., 2016). Consistent with these feeding preference attributes of bees, the highest abundance and widest variety of amino acids were found in the cotton floral nectar, which uniquely contained GABA, a non-proteinaceous amino acid.

When compared with the floral nectar, the extrafloral nectars, which act as rewards for mutualist ants, are characterized by higher concentrations of the disaccharide sucrose. The overall amino acid abundance and number of amino acids detected is lower in the extrafloral nectars, but the amino acid content is not dominated by a single amino acid. These characteristics likely reflect the feeding preferences of worker ants for dietary sources rich in carbohydrates (i.e., sucrose) and a complex mixture of amino acids (i.e. not dominated by a single amino acid) needed to provide a dietary source of nitrogen (Lanza, 1988; Blüthgen and Fiedler, 2004; Dussutour and Simpson, 2009).

### Nectary capacity for de novo amino acid synthesis and transport may contribute to nectar amino acid constituents

The expression patterns of the core nitrogen reduction and assimilation genes (reviewed by Dechorgnat et al., 2010) illustrate the potential capacity of cotton nectaries to biochemically reduce nitrate and incorporate the resulting ammonium into organic forms, such as glutamate and glutamine; this is particularly apparent in the floral and reproductive extrafloral nectaries (Figure 11). As supported by the parallel upregulated expression of genes associated with amino acid biosynthesis and transporters at the pre-secretory and secretory stages of nectary development, glutamate produced via ammonium assimilation would serve as the donor of the amine moiety for the biosynthesis of other amino acids. These amino acids would then undergo symplastic transport to the head cells of the papillae through the action of the upregulated amino acid transmembrane transporters, culminating in deposition into the secreted nectars.

While nitrogen assimilation from oxidized forms of nitrogen commonly occurs in roots, and this process also occurs in shoots where photosynthesis can provide the energy source (Meyer and Stitt, 2001; Lin et al., 2008), but few reports cite these processes as occurring in nectaries. For example, Solhaug et al. (2020) have reported the induction of nitrogen metabolism in *C. pepo*, and they surmised this occurs for amino acid biosynthesis and for nitric oxide production.

### Expression of *SWEET9* and *CWINV4* is predictive of nectar sugar profiles

The relative expression levels of *SWEET9* and *CWINV4* at the secretory stage of nectary development appear to be predictive of whether the nectar product will be hexose rich. As with Arabidopsis and pennycress (*Thlaspi arvense*) nectaries (Kram et al., 2009; Ruhlmann et al., 2010; Bender et al., 2012; Lin et al., 2014; Thomas et al., 2017), which produce hexose-rich nectars, at the secretory stage of cotton floral nectary development, the expression of *SWEET9* and *CWINV4* is near equal, and this nectary produces the most hexose-dominant nectar of the cotton nectaries. In contrast, the other three cotton nectaries produce nectars that are more sucrose enriched, and *CWINV4* expression is almost one-sixth the level of *SWEET9* expression (Figure 7 and Supplemental Dataset S2). Similarly, disproportionate expression of *SWEET9* and *CWINV4* has been reported in nectaries of pumpkin, squash, and sunflower, all of which produce sucrose-rich nectars (Chatt et al., 2018; Prasifka et al., 2018; Solhaug et al., 2019).

### Cell wall and cuticle alterations facilitate nectar release

Cotton nectar constituents are ultimately secreted from the papillae head cells, passing through the cell wall and cuticle. Our morphological and anatomical studies of reproductive extrafloral nectaries indicate that this passage is facilitated by microscopic physical alterations in the structure of the cell wall and cuticle. Consistent with the physical alterations of these polymeric barrier structures, the expression of genes encoding cell wall remodeling genes is upregulated, which likely contributes to the development of cell wall ingrowths observed on papillae head cells of the bracteal and circumbracteal nectaries. These ingrowths increase the surface area available for the secretion of nectar components (Fahn, 1979; Kronestedt et al., 1986; Paiva, 2016; Bartosz et al., 2018), which may be particularly important for the reproductive extrafloral nectaries that produce the largest volume of nectar and are active for the duration of fruit maturation (Wäckers and Bonifay, 2004).

Following passage through the cell wall, nectar initially accumulates in the subcuticular space between the cell wall and cuticle, as evident by the separation of the cuticle from the cell wall at the papillae head cells in all four cotton nectary types. In parallel, microchannels or fractures develop in the cuticle, potentially due to hydrostatic pressure, which would facilitate the release of nectar from the nectary. These actions may require the deposition of new cuticular lipids, which may be the driver for the observed upregulated expression of cuticle deposition genes. This phenomenon of cuticle separation and fracturing commonly occurs in the trichomatic nectaries of a variety of other species, included within Malvaceae (Findlay et al., 1971b; Kronestedt et al., 1986; Sawidis et al., 1987), and these cuticular channels would thus function as valves, releasing discrete droplets of nectar once hydrostatic pressure exceeds a threshold (Findlay et al., 1971b).

In summary, our combined systems-level studies of the expression of four different *G. hirsutum* nectaries have generated data that support a coordination between both the merocrine-based and eccrine-based models of nectar synthesis and secretion. Thus, this illustrates the complexity of the coordination among different cell populations to generate a functional nectary. Furthermore, the eccrine-based model was primarily developed from prior studies of eudicot floral nectaries. Therefore, this study has expanded the utility of the eccrine model to explain these complex processes as they occur in trichomatic extrafloral nectaries.

## Materials and methods

### Plant materials

Plants were grown in a Conviron Environmental Growth Chambers (0.7 m × 1.8 m × 1.4 m) that were kept in a cycle of 12 h illumination at 26°C starting at 6:00 local time, and 12 h darkness at 22°C. Seeds of *G. hirsutum* accession TM-1 were chipped and germinated in 8 cm × 8 cm × 10 cm pots filled with a soil mixture of 3-parts LC8 soil (www.sungro.com) to 1-part sand. Individual seedlings were transplanted into 2-gallon (2A) pots after reaching approximately 30 cm in height, and 10 g of Osmocote Fertilizer 19-5-8 (www.amleo.com) was mixed into the soil mixture per pot. Each growth chamber contained five plants. Plants were watered each day with tap water and once per week with a 10% fertilizer solution mixture of Scotts Excel 21-5-20 all-purpose water-soluble fertilizer and Scotts Excel 15-5-15 Cal-Mag water soluble fertilizer, obtained from a local garden shop. To ensure that this fertilizer application does not skew nectary function and nectar composition (Gijbels et al., 2015), all plants that were evaluated in this study were identically treated in these fertilizer applications.

### Collection of nectary and nectar samples

All nectary and nectar samples were collected from plants after the first flower bloomed, approximately 70 d after sowing. Nectar samples were collected between 10 AM and 3 PM local time, using a 5-µL Drummond Microdispenser (www.drummondsci.com). Nectar samples were first harvested before nectary tissue was excised using a sterile scalpel. Nectary samples were collected from leaves or flowers immediately after removal of each organ from the plants, and the collected nectary tissues were immediately flash-frozen in liquid nitrogen and stored at −80°C.

In this study, we analyzed four types of nectaries, the floral, bracteal, and circumbracteal nectaries collected from flowers, and foliar nectaries collected from leaves. The developmental trajectory of each nectary type was defined relative to nectar secretion and is defined as pre-secretory, secretory, and post-secretory stages. Thus, in the case of floral nectaries, these three developmental stages were collected at 24 h pre-anthesis, at anthesis, and at 24 h post-anthesis. The three equivalent developmental stages for bracteal and circumbracteal nectaries are defined as, (1) the “match-head square stage” of cotton square development (Main, 2012), (2) anthesis, and (3) 19–24 d after anthesis. Analogously, the three developmental stages of foliar nectaries were collected from leaves with a midvein length of 5–6 cm, a midvein length of 12–15 cm, and fully mature leaves that lacked visible nectar deposits.

### Non-targeted metabolomics analysis of nectar metabolites

Two separate GC–MS-based methods were employed for non-targeted metabolite profiling of nectar samples. Six replicate nectar samples were collected for each of the four nectar types. Each replicate consisted of pooled nectar, sampled from a minimum of three nectaries harvested from two plants on a single day.

One of these analysis methods provided data on the predominant sugars that constitute the nectar (i.e. sucrose, glucose, and fructose). Specifically, 1 µL of nectar, spiked with an internal standard (10 µg ribitol) was dried by lyophilization. The sample was methoximated at 30°C for 90 min, while continuously shaking with 20 mg mL^−1^ methoxyamine hydrochloride dissolved in pyridine. The methoximated sample was silylated for 30 min at 60°C with *N*,*O*-Bis(trimethylsilyl)trifluoroacetamide and 1% w/v trimethylchlorosilane. Following dilution with 1.5 mL pyridine, 1 µL sample was analyzed by GC–MS. GC parameters were set to a helium gas flow rate of 1 mL min^−1^, 1 µL injection with a 10:1 split, and a temperature gradient of 100°C–180°C increasing at a rate of 15°C min ^−1^, then 5°C min ^−1^ to 305°C, then 15°C min ^−1^ to 320°C, followed by a 5-min hold at 320°C.

The second analysis method focused on the less abundant constituents of the nectar, which were extracted from a 5-µL aliquot of nectar sample that was spiked with 0.5 µg nonadecanoic acid and 1 µg ribitol, as internal standards. Hot methanol (2.5 mL) was added to the nectar and the mixture was incubated at 60°C for 10 min. Following sonication for 10 min at 4°C, chloroform (2.5 mL) and water (1.5 mL) were sequentially added, and the mixture was vortexed. Centrifugation at 4,000 rpm for 7 min at room temperature separated the polar and non-polar fractions, and the entire non-polar fraction and half of the polar fraction were recovered to separate 2 mL screw-cap glass vials and dried by lyophilization. The polar fraction underwent methoximation as previously described, and both polar and non-polar fraction were silylated for 30 min at 60°C with *N*,*O*-Bis(trimethylsilyl)trifluoroacetamide and 1% trimethylchlorosilane.

The derivatized metabolites (the sugars, polar, and non-polar fractions) were analyzed using an Agilent Technologies Model 7890A GC system equipped with an HP-5ms (30 m, 0.25 mm, 0.25 µm) column that was coupled to an Agilent Technologies 7683B series injector and Agilent Technologies Model 5975C inert XL MSD with Triple-Axis Detector mass spectrometer (www.agilent.com). Chromatography parameters for the polar and non-polar fractions were set to a helium gas flow rate of 1 mL min^−1^, 2 µL injection, with a temperature gradient of 80°C–320°C increasing at a rate of 5°C min ^−1^, followed by a 9-min hold at 320°C. The polar fractions were analyzed using a “heart-cut” method which diverted gas flow to an FID detector during elution times for fructose, glucose, and sucrose. Deconvolution and integration of resulting spectra were performed with Automated Mass Spectral Deconvolution and Identification System (AMDIS) software (Stein, 1999). Analyte peaks were identified by comparing mass spectra and retention indices to the NIST14 Mass Spectral Library (https://chemdata.nist.gov/) and authentic standards when possible to confirm identification.

### Amino acid analysis

Analysis of amino acids was performed using the Phenomenex EZ:Faast kit for free amino acids (www.phenomenex.com). Six replicate samples for each nectar type were collected as described previously. Due to low volume of nectar produced by the foliar nectary, these nectar samples were pooled from a maximum of 90 nectaries, collected from six separate plants. Each sample (20 µL nectar per extraction) was subjected to solid phase extraction and derivatized according to the manufacturer’s instructions, with one adjustment: after addition of the norvaline internal standard (5 nmol) to each sample, 125 µL of 10% propanol (v/v)/20 mM HCl was added to acidify the sample. Following derivatization, samples were concentrated by evaporation under a stream of nitrogen gas before amino acids were analyzed using an Agilent Technologies model 6890 gas chromatograph with a ZB-AAA 10 m × 0.25 mm amino acid analysis column coupled to a model 5973 mass selective detector capable of electrical ionization (EI). The GC–MS instrument settings followed the manufacturer’s recommendations.

### Statistical analysis of cotton nectar metabolites

For each metabolite, the natural logarithm of normalized metabolite level was averaged over the six replicates for each nectar type. Separately for each metabolite, a linear model with one mean per species and constant error variance was fitted to the metabolite response values. As part of each linear model analysis, *F*-tests for contrasts among the four nectar type means were conducted to identify differences in average response between each pair of nectar types. The 197 *P*-values for each comparison (one *P*-value per metabolite) were adjusted to obtain approximate control of the false discovery rate at the 0.05 level (Benjamini and Hochberg, 1995; Nettleton et al., 2006).

Similarities and differences among metabolites between different nectary types were visualized by pair-wise volcano plot comparisons and hierarchical agglomerative clustering. To perform clustering, the estimated nectar type response means were first standardized within each metabolite to obtain a standardized response profile across nectar types for each metabolite. Then dissimilarity between each pair of metabolites was computed as the Euclidean distance between the standardized response profiles. Clustering based on these pairwise dissimilarities places two metabolites in the same cluster if their estimated nectar type response means are highly correlated across sections. Although hierarchical clustering groups the metabolites into any number of clusters, a total of 16 clusters were selected to display and summarize the results, striking a balance between high within-cluster consistency and low between-cluster similarity.

### Light microscopy and histochemistry

Pre-secretory and secretory stage nectaries were fixed for several days at 4°C, in a solution of 3% (w/v) glutaraldehyde and 2% (w/v) paraformaldehyde in 0.1 M sodium cacodylate buffer, pH 7.2. Samples were dehydrated in a graded ethanol series (50%–100%), followed by infiltration and embedding over 5 d in LR White resin. For replication purposes, a minimum of four nectaries per nectary type was imbedded at each developmental stage. Resin blocks were polymerized at 55°C for 72 h. Histological sections were cut at 1.3-µm thickness using a Leica UC6 Ultramicrotome (www.leica-microsystems.com). Sections were dyed with toluidine blue O for general contrast and PAS technique for starch and other non-water-soluble carbohydrates (Ruzin, 1999). Digital images were collected using a Zeiss Axiocam HRC camera (www.zeiss.com) on an Olympus BX-40 compound microscope (www.olympus-ims.com) in bright-field mode.

### Transmission electron microscopy

A minimum of four nectaries, of the four nectary types (foliar, bracteal, circumbracteal, and floral), harvested at the secretory stage, were fixed for several days at 4°C, in a solution of 3% (w/v) glutaraldehyde and 2% (w/v) paraformaldehyde in 0.1 M sodium cacodylate buffer, pH 7.2. Samples were washed with several changes of 0.1 M sodium cacodylate buffer, pH 7.2, and then fixed in 1% osmium tetroxide in 0.1 M sodium cacodylate buffer for 1 h at room temperature. The samples were *en block* stained for 2 h with aqueous 2% (w/v) uranyl acetate, and then dehydrated in a graded ethanol series (50%–100%). Following a transition into ultra-pure acetone, and infiltrating, the nectaries were embedded with Spurr’s hard epoxy resin (www.emsdiasum.com). Resin blocks were polymerized for 48 h at 70°C. Thick sections (1 µm) to check fixation quality and ultrathin (90 nm) sections were made using a Leica UC6 ultramicrotome (www.leica-microsystems.com). Ultrathin sections were collected onto carbon-film, single-slot copper grids and images were captured using a JEM 2100 200 kV scanning and transmission electron microscope (www.jeol.com).

### Scanning electron microscopy

A minimum of four nectaries per nectary type and at the pre-secretory and secretory stages of development were fixed for several days at 4°C in formalin–acetic acid–alcohol (5% v/v, 5% v/v, and 50% v/v, respectively). They were dehydrated in a graded ethanol series (50% v/v, 70, 95, 100, 100 ultra-pure twice). Samples were critical point-dried using a Denton Drying Apparatus, Model DCP-1 (www.dentonvacuum.com). The dried specimens were mounted on aluminum stubs with 12-mm circular carbon adhesive tabs and colloidal silver paint (www.emsdiasum.com). Samples were sputter coated with 30 nm platinum using a Cressington HR208 Sputter Coater (www.cressington.com). Images were captured using a Hitachi SU-4800 field emission SEM at 10 kV (www.hitachi-hightech.com).

### RNA isolation, sequencing, and informatics

Triplicate RNA samples were isolated for each of the nectary types. Each replicate was a pool of approximately 2–4 floral or 10–15 of each of the extrafloral nectaries. Tissue was transferred with clean forceps into a 2-mL Lysing matrix A tube (MP Biomedicals; Ref. No. 6910-500; www.mpbio.com), resting in a liquid nitrogen bath and containing a ceramic bead. The tubes were quickly transferred to a QuickPrep adaptor (containing dry ice) and attached to the FastPrep 24-5G (www.mpbio.com) benchtop homogenizer for tissue pulverization. The samples were subjected to five to six pulverization cycles of 40 s each, at 6 m/s, with each cycle interjected with a period of immersion in liquid nitrogen and refilling the adaptor with dry ice. Post-pulverization, 600 µL of the RNA lysis buffer of the Quick-RNA MiniPrep kit (Zymo Research; Cat. No. R1054; www.zymoresearch.com) was quickly added to the Lysing matrix tube and the tubes were vortexed. This was followed by the addition of 50 µL of the Plant RNA Isolation Aid (Thermo Fisher Scientific, Cat. No. AM9690; erstwhile Ambion) to remove common plant contaminants such as polyphenolics and polysaccharides. Quick-RNA MiniPrep kit directions were followed for RNA isolation. Agarose gel electrophoresis and UV spectrophotometry were used to assess RNA quality, prior to submission to the University of Minnesota Genomics Center for barcoded cDNA library creation and Illumina HiSeq 2500 sequencing. This produced over 360 million 125-bp paired-end reads with a target insert size of 200 bp and generated ≥24 M reads for each sample, and the average quality scores were above Q30. A few samples did not yield suitable sequencing libraries and thus were omitted from the analysis.

The reads were mapped to the UTX-JGI *G. hirsutum* genome (v1.1) and predicted transcripts using NCBI’s BLASTN (Camacho et al., 2009). The UTX-JGI annotation was used to map read counts to Arabidopsis genes (Araport 11). Read counts were upper-quartile normalized and pairwise differential expression tests were performed using a negative binomial distribution with DESeq (Anders and Huber, 2010). The resulting *P*-values were filtered by restricting to genes with a 50% or greater change in mean normalized counts. The Benjamini–Hochberg method was used to control the false discovery rate at the 0.05 level (Benjamini and Hochberg, 1995).

Differentially expressed genes were identified by filtering the DESeq results within R and categorized (e.g., upregulated during the secretory stage); these categories were visualized by generating Venn diagrams using InteractiVenn (Heberle et al., 2015). GO enrichment analysis of the nectary transcriptome was implemented using topGO: Enrichment Analysis for GO (https://bioconductor.org/packages/release/bioc/html/topGO.html) with prior gene-to-GO term mapping completed using GO.db (https://bioconductor.org/packages/GO.db/). A Fisher’s exact test was completed to test for enrichment of GO terms in specific expression pattern groups, using the complete set of 16,958 Arabidopsis orthologs as the baseline for this comparison. Redundancy in the resulting lists of enriched GO terms was removed using REVIGO (Supek et al., 2011).

Mapping genes to metabolic pathways used MapMan (Thimm et al., 2004) with the base pathways and mappings files for Arabidopsis. Hierarchical clustering based on one minus Pearson correlation of the log_2_ normalized read count of selected metabolic pathways or functionalities was completed using Morpheus (https://software.broadinstitute.org/morpheus).

### Reverse transcription quantitative PCR validation

The same RNA samples used for RNA-seq analyses were used as the template for cDNA synthesis using the BioRad iScript cDNA synthesis kit (Catalog No. 1708890), with 1 μg of RNA used for cDNA preparation. Expression patterns for representative genes that displayed stage-specific variation via RNAseq analyses were validated by RT‐qPCR using Agilent Brilliant III Ultra‐fast SYBR Green QPCR Master Mix (Catalog No. 600882) and a final cDNA template concentration of 2 ng/μL. Relative expression values are presented as linearized ΔCt (2^ΔCt^) values normalized to the reference gene (Schmittgen and Livak, 2008). Gene expression was normalized to the reference gene encoding a 40S ribosomal protein S3-2-like gene (Cotton gene ID = Gohir.D05G034300.1, 1). This gene was chosen as the internal reference based on its stable expression level in the RNA-seq dataset collected from floral and bracteal nectary samples. Primer sequences for each gene are provided in Supplemental Dataset S10.

## Accession numbers

Raw sequence reads are available at the National Center for Biotechnology Information Sequence Read Archive under GEO accession number GSE113373. Metabolomics data are publicly available in the PMR database (http://metnetweb.gdcb.iastate.edu/PMR/).

## Supplemental data

**Supplemental Figure S1.** Venn diagram representation of the *G. hirsutum* nectar metabolomes.

**Supplemental Figure S2.** Hierarchical clustering analysis by nectar type of the 197 quantified nectar analytes.

**Supplemental Figure S3.** Amino acid profiles of *G. hirsutum* nectars categorized as non-proteinaceous, essential, and non-essential amino acids.

**Supplemental Figure S4.** Light micrographs of longitudinal sections of different *G. hirsutum* nectaries stained with toluidine blue O for general morphology.

**Supplemental Figure S5.** GO enrichment analysis of genes identified in Figure 7, C, as uniquely up- or down-regulated during the secretory stage of development in each nectary type.

**Supplemental Figure S6.** Heat map representation of temporal differential expression of genes annotated as transmembrane transporters.

**Supplemental Figure S7.** Heat map representation of temporal differential expression of genes annotated as involved in cell wall metabolism.

**Supplemental Figure S8.** Heat map representation of temporal differential expression of genes annotated as involved in lipid metabolism.

**Supplemental Table S1.** Morphological and anatomical structural comparisons among nectaries

**Supplemental Dataset S1.** Summary of nectar metabolomics results and analyses to include hierarchical clustering.

**Supplemental Dataset S2.** Normalized reads for all biological replicates, including DESeq analyses.

**Supplemental Dataset S3.** Normalized reads and DESeq analyses for genes displaying nectary tissue preferential expression, but not modulated by the developmental stage of each nectary.

**Supplemental Dataset S4.** Listing of normalized reads and DESeq analyses for genes displaying nectary tissue preferential expression, but not modulated by the developmental stage of each nectary.

**Supplemental Dataset S5.** GO term enrichment analysis of genes differentially expressed between nectary and non-nectary tissues.

**Supplemental Dataset S6.** Normalized reads and DESeq analyses for genes that are differentially expressed in each nectary type, and expression is also modulated by the development of each nectary type. Data are organized by nectary type and gene expression pattern.

**Supplemental Dataset S7.** Normalized reads and DESeq analyses for genes that are differentially expressed in each nectary type, and expression is also modulated by the development of each nectary type. Each tab is specific to a gene expression patterns normalized to the secretory developmental stage and lists the genes present within each section of the Venn diagrams displayed in Figure 8, C.

**Supplemental Dataset S8.** GO term enrichment analysis of genes differentially expressed in each nectary type, and expression is also modulated by the development of each nectary type as presented by the Venn diagram of Figure 8, C.

**Supplemental Dataset S9.** Annotation of cotton nectary transcriptomes based on metabolic pathway or function using MapMan and GO terms.

**Supplemental Dataset S10.** Primer sequences for RT-qPCR.

## Supplementary Material

kiab018_Supplementary_Data

## Notes

E.C.C., C.J.C., and B.J.N. conceived and planned the study. E.C.C., S.-N.M., and N.-A.M.-F. conducted sample collection, microscopy, and metabolomic analyses. Additional contributions for microscopic analyses were provided by H.T.H. P.M.K., R.R., and C.J.C. completed the RNA preparation and RT-qPCR analysis. M.H. processed all RNAseq data and conducted informatics analyses in conjunction with E.C.C. E.C.C. and B.J.N. wrote the manuscript with feedback from all coauthors.

The author responsible for distribution of materials integral to the findings presented in this article in accordance with the policy described in the Instructions for Authors (https://academic.oup.com/plphys/pages/general-instructions) is: Basil J. Nikolau (dimmas@iastate.edu).
