# Supplementary material for: Nectar biosynthesis is conserved among floral and extrafloral nectaries
Source: Plant Physiol. 2021 Jan 28;185(4):1595–616. doi: 10.1093/plphys/kiab018 (PMC8133665; doi:10.1093/plphys/kiab018)
Supplement: kiab018_Supplementary_Data [file kiab018_supplementary_data.zip › pp.01245.2020-s02.docx]

| **Supplemental Table S1 \|** Morphological and anatomical structural comparisons among one floral (FL) and three extrafloral [Bracteal (B), Circumbracteal (C), and Foliar (FO)] cotton nectaries at pre-secretory (P) and secretory (S) stages. Red text highlights the differences among nectaries. | | | | | | | | |
| --- | --- | --- | --- | --- | --- | --- | --- | --- |
| **Structural Detail** | **Pre-FL** | **Sec-FL** | **Pre-B** | **Sec-B** | **Pre-C** | **Sec-C** | **Pre-FO** | **Sec-FO** |
| Distribution of starch within subnectariferous parenchyma | Towards proximal portion and near vascular bundles | Towards proximal portion and near vascular bundles | Towards proximal portion and near vascular bundles | Absent near vascular bundles | Towards proximal portion and near vascular bundles | Absent near vascular bundles | Absent | Absent |
| Distribution of phenolic bodies | Abundant and large near vascular bundles; smaller within nectariferous parenchyma | Abundant and large near vascular bundles; smaller within nectariferous parenchyma | Abundant and large near vascular bundles; smaller within nectariferous parenchyma | Abundant and large near vascular bundles; smaller within nectariferous parenchyma | Abundant and large near vascular bundles; smaller within nectariferous parenchyma | Abundant and large near vascular bundles; smaller within nectariferous parenchyma | Abundant and large near vascular bundles; smaller within nectariferous parenchyma | Abundant and large near vascular bundles; smaller within nectariferous parenchyma |
| Layers of subnectariferous parenchyma | ~10 layers | ~10 layers | ~10 layers | ~10 layers | ~10 layers | ~10 layers | ~10 layers | ~10 layers |
| Vascular bundles near subnectariferous parenchyma | Yes | Yes | Yes | Yes | Yes | Yes | Yes-phloem rays extend into nectariferous parenchyma | Yes- phloem rays extend into nectariferous parenchyma |
| Nectariferous parenchyma isodiametric small intercellular spaces, dense-staining cytoplasm | Yes | Yes | Yes | Yes | Yes | Yes | Yes | Yes |
| Layers of nectariferous parenchyma | 3-4 proximal, 2 distal | 3-4 proximal, 2 distal | 3-4 | 3-4 | 3-4 | 3-4 | 3-4 | Up to 6 |

| **Structural Detail** | **Pre-FL** | **Sec-FL** | **Pre-B** | **Sec-B** | **Pre-C** | **Sec-C** | **Pre-FO** | **Sec-FO** |
| --- | --- | --- | --- | --- | --- | --- | --- | --- |
| Druses in subnectariferous parenchyma and around vascular bundles | Yes-extend in files to papillae, most abundant | Yes-extend in files to papillae, most abundant | Yes-extend in files to papillae | Yes-extend in files to papillae | Yes-extend in files to papillae | Yes-extend in files to papillae | Yes-extend in files to papillae | Yes-extend in files to papillae |
| Epidermis bordering papillae | Highly vacuolated, contains phenolic bodies | Highly vacuolated, contains phenolic bodies | Highly vacuolated | Highly vacuolated | Highly vacuolated | Highly vacuolated | Highly vacuolated, contains phenolic bodies | Highly vacuolated |
| Hypoepidermis below papillae | Dense cytoplasm and phenolic bodies | Dense cytoplasm and phenolic bodies | Vacuolate and phenolic bodies | Dense cytoplasm and phenolic bodies | Vacuolate and phenolic bodies | Dense cytoplasm and phenolic bodies | Vacuolate and phenolic bodies | Dense cytoplasm and phenolic bodies |
| Nectary papillae multicellular with three regions | Yes | Yes | Yes | Yes | Yes | Yes | Yes | Yes |
| Number of papillae cell  layers | <12-14 | 12-14 | <5-6 | 5-6 | <5-6 | 5-6 | <5-6 | 5-6 |
| Number of basal cells, less electron dense | 2 | 2 | 1 | 1 | 1 | 1 | 1 | 1 |
| Stalk cell features | Phenolic bodies, vacuolate | Phenolic bodies, highly vacuolate, vacuole size decreases but number increases | Phenolic bodies around cell periphery, no vacuoles | Phenolic bodies around cell periphery, highly vacuolated | Phenolic bodies around cell periphery, no vacuoles | Phenolic bodies around cell periphery, highly vacuolated | Phenolic bodies, vacuolate | Phenolic bodies, highly vacuolated, vacuole size decreases but number increases |
| Head cell vacuoles, cell walls, and cuticle | Vacuoles, cuticle attached to cell wall | Highly vacuolated, cuticle separates from cell walls and forms surface microchannels | No vacuoles,  cuticle partially separated from walls | Highly vacuolated, cuticle separates from cell walls and forms surface microchannels | No vacuoles,  cuticle partially separated from walls | Highly vacuolated, cuticle separates from cell walls and forms surface microchannels | Vacuoles | Highly vacuolated, cuticle separates from cell walls and forms surface microchannels |
| **Structural Detail** | **Pre-FL** | **Sec-FL** | **Pre-B** | **Sec-B** | **Pre-C** | **Sec-C** | **Pre-FO** | **Sec-FO** |
| Papillae cuticle and cell walls | Thinnest around head cells and thickest around basal cells | Thinnest around head cells and thickest around basal cells | Thinnest around head cells and thickest around basal cells | Thinnest around head cells and thickest around basal cells, cell wall ingrowths, periplasmic space | Thinnest around head cells and thickest around basal cells | Thinnest around head cells and thickest around basal cells, cell wall ingrowths | Thinnest around head cells and thickest around basal cells | Thinnest around head cells and thickest around basal cells |
| Mitochondria, highest number in basal cells | N/A | Yes | N/A | Yes | N/A | Yes | N/A | Yes |
| RER, highest number in basal cells | N/A | Yes | N/A | Yes | N/A | Yes | N/A | Yes |
| Vesicles often observed fused with plasma membranes | N/A | Yes | N/A | Yes | N/A | Yes | N/A | Yes |
| Amyloplasts | N/A | Present | N/A | Present | N/A | Present | N/A | Present |
| Chloroplasts | N/A | Absent | N/A | Present | N/A | Absent | N/A | Present |
| Golgi bodies | N/A | Present | N/A | Present | N/A | Present | N/A | Present |
| Plasmodesmata observed in all walls of papillae and nectariferous parenchyma | N/A | Y | N/A | Y | N/A | Y | N/A | Y |
