# Supplementary material for: Nectar biosynthesis is conserved among floral and extrafloral nectaries
Source: Plant Physiol. 2021 Jan 28;185(4):1595–616. doi: 10.1093/plphys/kiab018 (PMC8133665; doi:10.1093/plphys/kiab018)
Supplement: kiab018_Supplementary_Data [file kiab018_supplementary_data.zip › pp.01245.2020-s12.pdf]

Supplemental Figures
Chatt *et al.*, Cotton floral and extrafloral nectaries

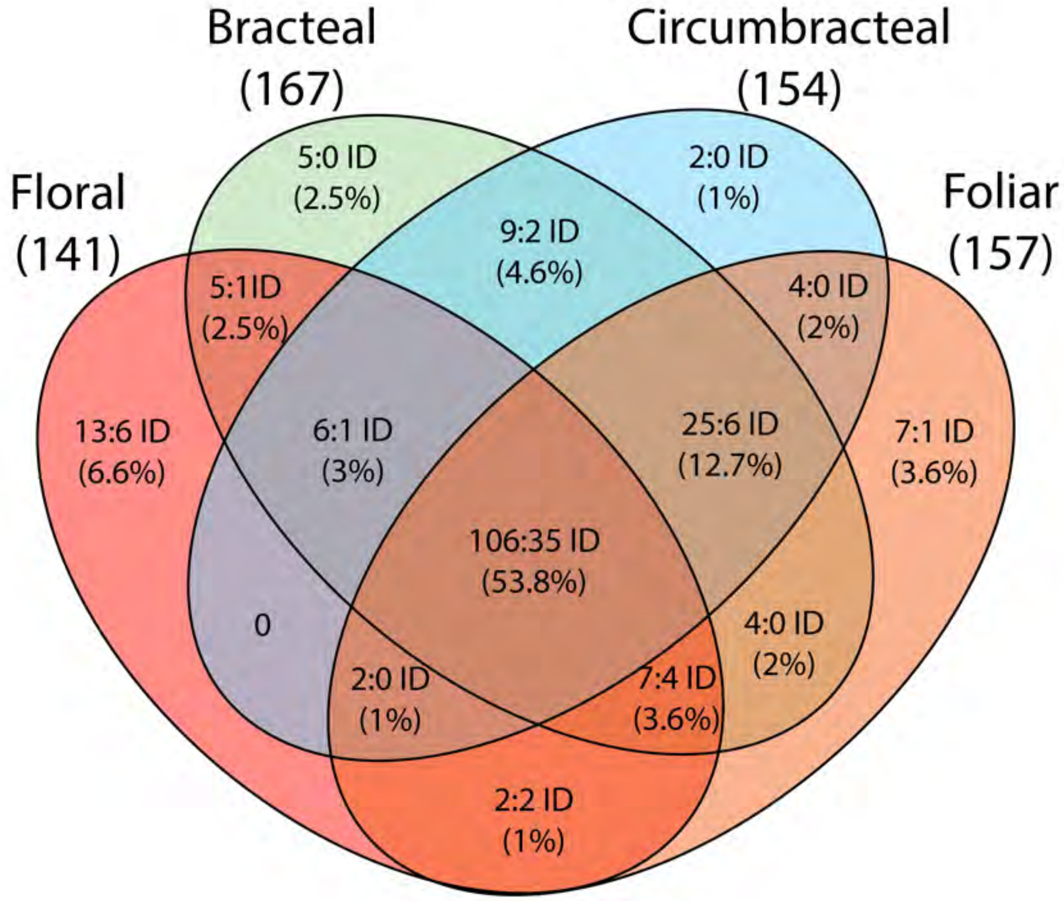


SUPPLEMENTAL FIGURE S1 | Venn diagram representation of the *G. hirsutum* nectar metabolomes. Each set is labeled with the number and percentage of analytes falling into that set along with the number of chemically identified analytes (the number after the colon).

Supplemental Figures
Chatt *et al.*, Cotton floral and extrafloral nectaries

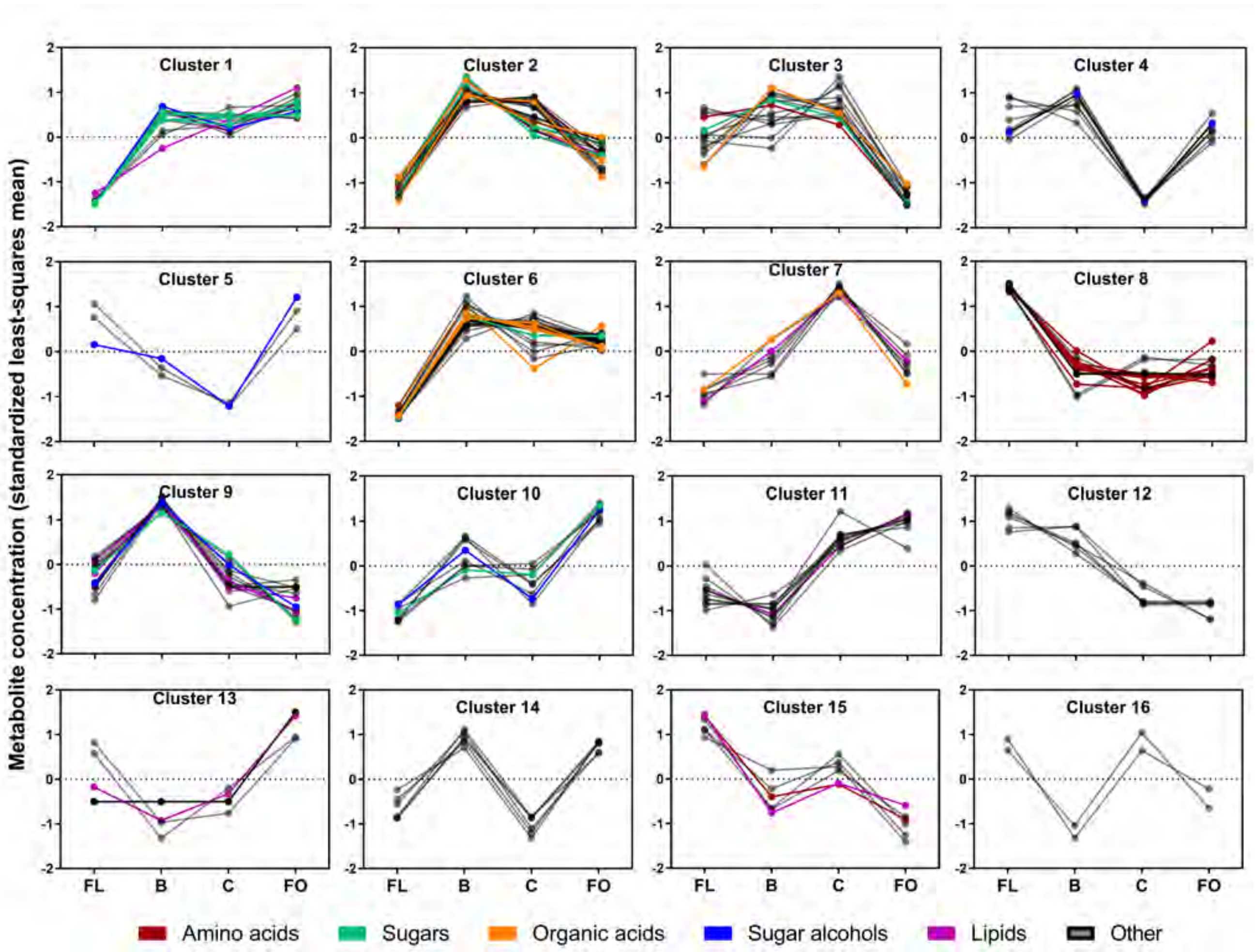


SUPPLEMENTAL FIGURE S2 | Hierarchical clustering analysis by nectar type of the 197 quantified nectar analytes. Abbreviations: FL = floral; B = bracteal; C = circumbracteal; FO = foliar.

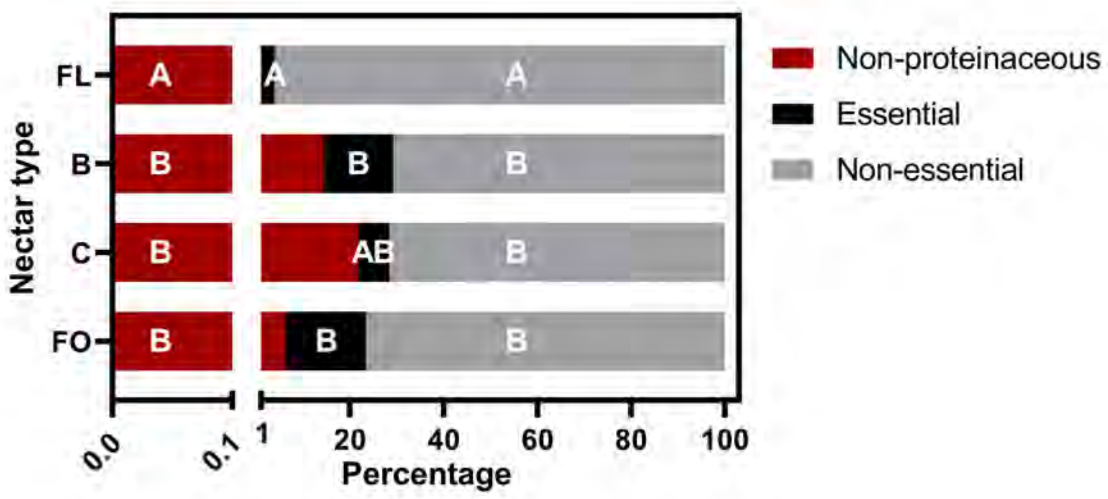


SUPPLEMENTAL FIGURE S3 | Amino acid profiles of *G. hirsutum* nectars catego-rized as non-proteinaceous, essential, and non-essential amino acids. Essential amino acids include leucine, isoleucine, phenylalanine, threonine, tryptophan, and valine. Letters on each data bar indicate statistical significance in abundance (q-value < 0.05). Abbreviations: FL = floral; B = bracteal; C = circumbracteal; FO = foliar.

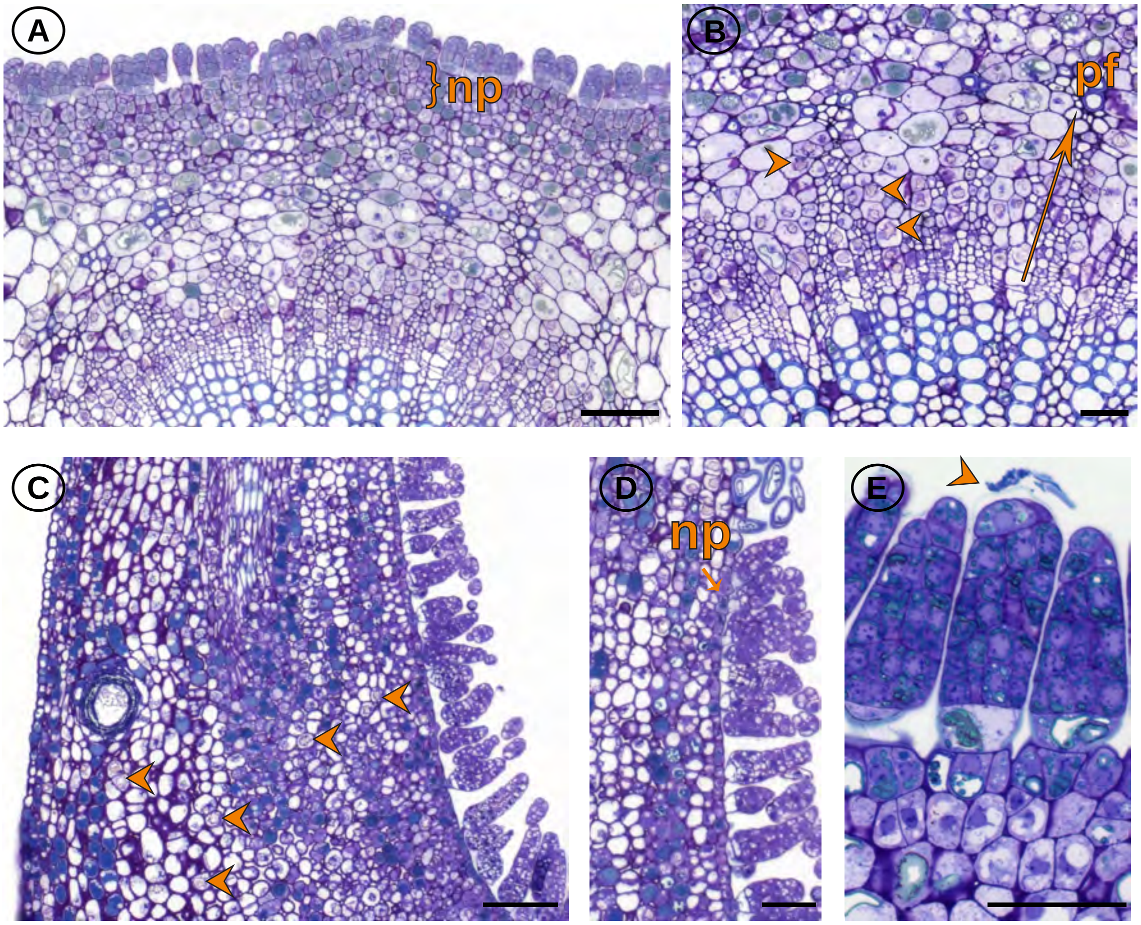


SUPPLEMENTAL FIGURE S4 | Light micrographs of longitudinal sections of different *G. hirsutum* nectaries stained with Toluidine Blue O for general morphology. (A) Foliar secretory nectary overview; (B) Foliar secretory nectary, phloem rays extending into the subnectariferous parenchyma highlighted by arrow, arrow heads point to druse crystals; (C) Floral secretory nectary overview, arrow heads point to druse crystals; (D) Distal portion of floral secretory nectary highlighting the nectariferous parenchyma; (E) Pre-secretory bracteal nectary displaying separation of cuticle from head cells highlighted by arrow head. Abbreviations: np = nectariferous parenchyma; pf = phloem fiber. Scale bars A, C= 100 µm; B,D = 50 µm.

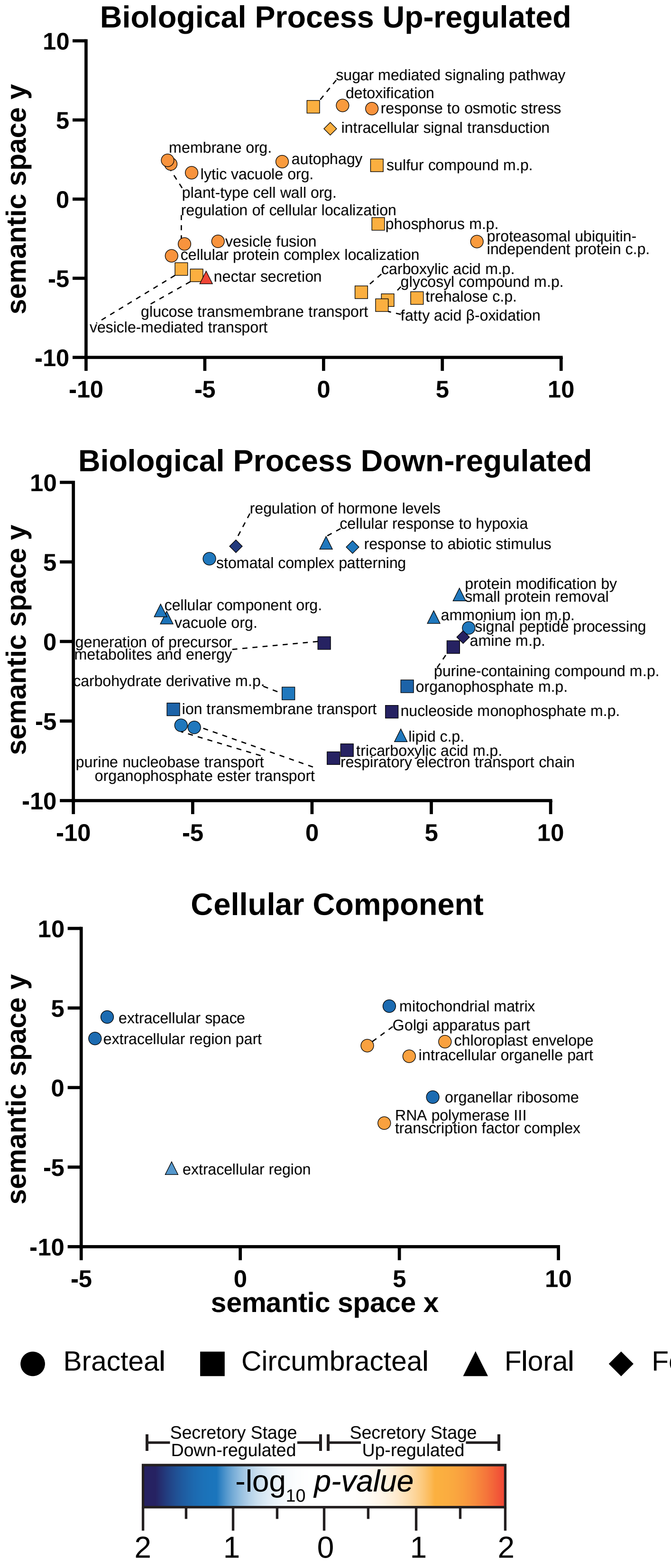


SUPPLEMENTAL FIGURE S5 | Gene ontology enrichment analysis of genes identified in Figure 8C, as uniquely up- or down-regulated during the secretory stage of development in a each nectary type. GO terms are clustered by the REVIGO visualization algorithm based on semantic similarity (semantic space). Details of the results are provided in Supplemental File 9. Abbreviations: m.p. = metabolic process; c.p. = catabolic process; org. = organization; t.t. = transmembrane transporter.

Supplemental Figures
Chatt *et al.*, Cotton floral and extrafloral nectaries

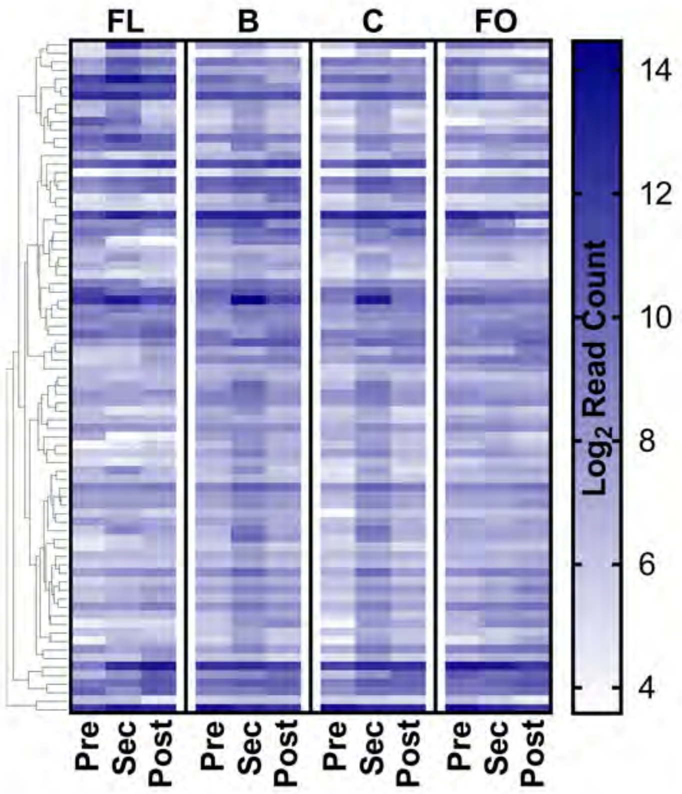


SUPPLEMENTAL FIGURE S6 | Heat map representation of temporal differential expression of genes annotated as transmembrane transporters. Genes were filtered by the characteristic of being upregulated in nectary tissue as compared to the non-nectary control tissue. Temporal differential expression was evaluated relative to the secretary stage nectaries. Genes were hierarchically clustered based on one minus Pearson correlation of the $log_2$ transformed normalized read counts. Gene descriptions are provided in Supplemental Dataset S9. Abbreviations: FL = floral; B = bracteal; C = circumbracteal; FO = foliar; Pre = pre-secretory; Sec = secretory; Post = post-secretory.

Supplemental Figures
Chatt *et al.*, Cotton floral and extrafloral nectaries

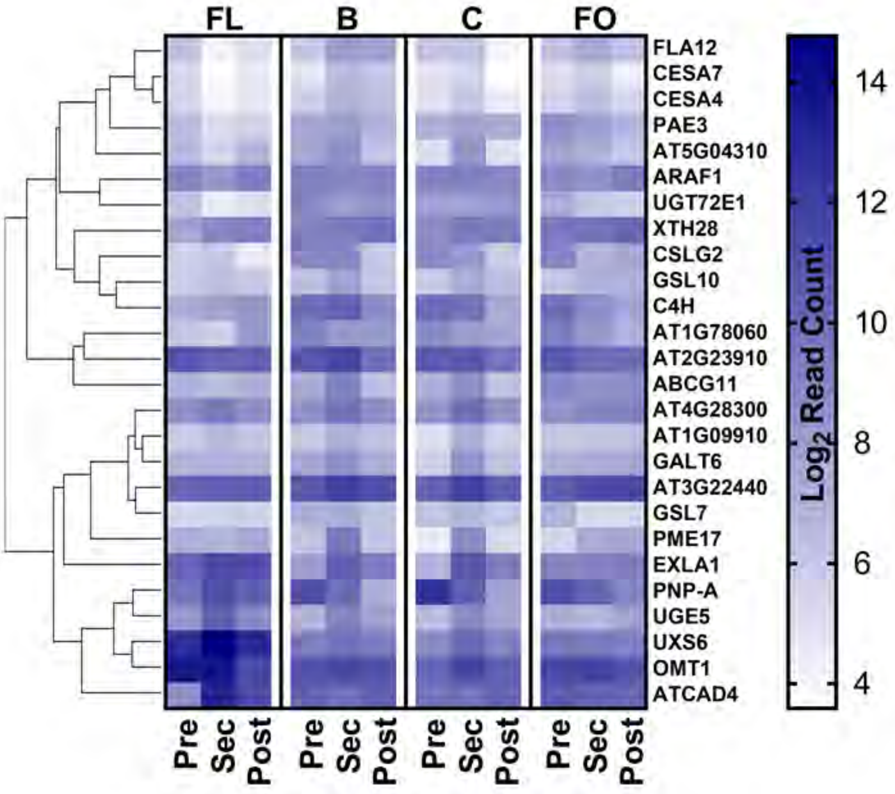


SUPPLEMENTAL FIGURE S7 **|** Heat map representation of temporal differential expression of genes annotated as involved in cell wall metabolism. Genes were filtered by the characteristic of being upregulated in nectary tissue as com-pared to the non-nectary control tissue. Temporal differential expression was evaluated relative to the secretary stage nectaries. Genes were hierarchically clustered based on one minus Pearson correlation of the $log_2$ transformed normalized read counts. Gene descriptions are provided in Supplemental File 10. Abbreviations: FL = floral; B = bracteal; C = circumbracteal; FO = foliar; Pre = pre-secretory; Sec = secretory; Post = post-secretory.

Supplemental Figures
Chatt *et al.*, Cotton floral and extrafloral nectaries

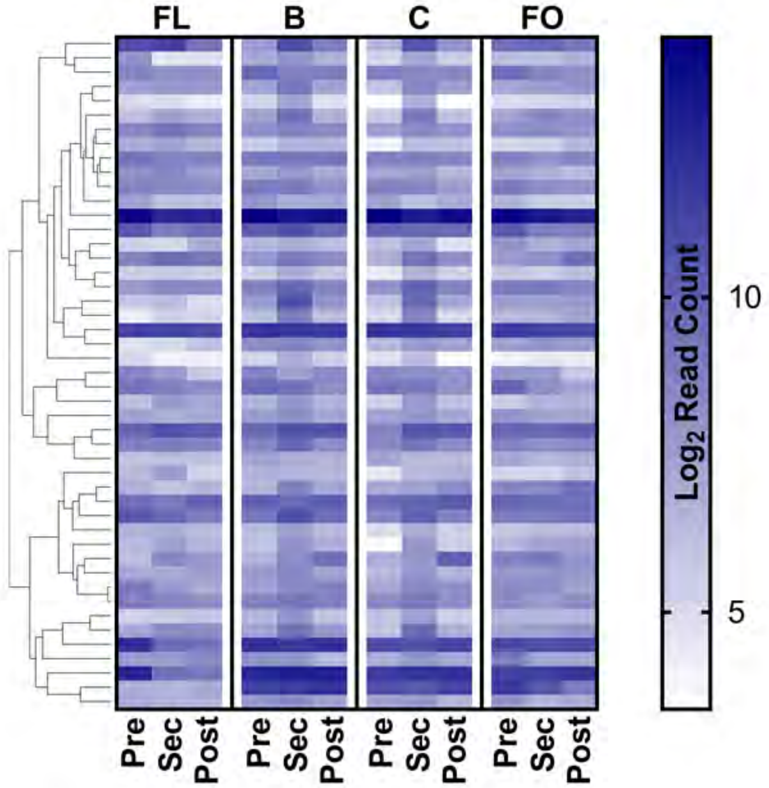


SUPPLEMENTAL FIGURE S8 | Heat map representation of temporal differential expression of genes annotated as involved in lipid metabolism. Genes were filtered by the characteristic of being upregulated in nectary tissue as compared to the non-nectary control tissue. Temporal differential expression was evaluated relative to the secretary stage nectaries. Genes were hierarchically clustered based on one minus Pearson correlation of the $\log_2$ transformed normalized read counts. Gene descriptions are provided in Supplemental File 10. Abbreviations: FL = floral; B = bracteal; C = circumbracteal; FO = foliar; Pre = pre-secretory; Sec = secretory; Post = post-secretory.
